# Supplementary material for: Broader impacts of an intervention to transform school environments on student behaviour and school functioning: post hoc analyses from the INCLUSIVE cluster randomised controlled trial
Source: BMJ Open. 2020 May 15;10(5):e031589. doi: 10.1136/bmjopen-2019-031589 (PMC7232400; doi:10.1136/bmjopen-2019-031589)
Supplement: Author's manuscript [file bmjopen-2019-031589.draft_revisions.pdf]

BMJ Open

BMJ Open is committed to open peer review. As part of this commitment we make the peer review history of every article we publish publicly available.

When an article is published we post the peer reviewers' comments and the authors' responses online. We also post the versions of the paper that were used during peer review. These are the versions that the peer review comments apply to.

The versions of the paper that follow are the versions that were submitted during the peer review process. They are not the versions of record or the final published versions. They should not be cited or distributed as the published version of this manuscript.

BMJ Open is an open access journal and the full, final, typeset and author-corrected version of record of the manuscript is available on our site with no access controls, subscription charges or pay-per-view fees (http://bmjopen.bmj.com).

If you have any questions on BMJ Open's open peer review process please email info.bmjopen@bmj.com

BMJ Open

Broader impacts of an intervention to transform school environments: additional analyses from the INCLUSIVE cluster randomised controlled trial

Journal:	*BMJ Open*
Manuscript ID	bmjopen-2019-031589
Article Type:	Original research
Date Submitted by the Author:	10-May-2019

Complete List of Authors:	Bonell, Christopher; London School of Hygiene and Tropical Medicine, Public Health and Policy<br>Dodd, Matthew; London School of Hygiene and Tropical Medicine, Public Health and Policy<br>Allen, Elizabeth; London School of Hygiene & Tropical Medicine, Department of Medical Statistics<br>Bevilacqua, Leonardo; University College London<br>McGowan, Jennifer; University College London<br>Opondo, Charles; London School of Hygiene and Tropical Medicine<br>Sturgess, Joanna; London School of Hygiene and Tropical Medicine, Department of Population Health<br>Elbourne, Diana; LSHTM, EPH<br>Warren, Emily; London School of Hygiene & Tropical Medicine, Faculty of Epidemiology and Population Health<br>Viner, Russell; UCL Institute of child health, Population, policy and practice research programme

Keywords:	PUBLIC HEALTH, EPIDEMIOLOGY, STATISTICS & RESEARCH METHODS

SCHOLARONE™
Manuscripts

I, the Submitting Author has the right to grant and does grant on behalf of all authors of the Work (as defined in the below author licence), an exclusive licence and/or a non-exclusive licence for contributions from authors who are: i) UK Crown employees; ii) where BMJ has agreed a CC-BY licence shall apply, and/or iii) in accordance with the terms applicable for US Federal Government officers or employees acting as part of their official duties; on a worldwide, perpetual, irrevocable, royalty-free basis to BMJ Publishing Group Ltd ("BMJ") its licensees and where the relevant Journal is co-owned by BMJ to the co-owners of the Journal, to publish the Work in this journal and any other BMJ products and to exploit all rights, as set out in our licence.

The Submitting Author accepts and understands that any supply made under these terms is made by BMJ to the Submitting Author unless you are acting as an employee on behalf of your employer or a postgraduate student of an affiliated institution which is paying any applicable article publishing charge ("APC") for Open Access articles. Where the Submitting Author wishes to make the Work available on an Open Access basis (and intends to pay the relevant APC), the terms of reuse of such Open Access shall be governed by a Creative Commons licence – details of these licences and which Creative Commons licence will apply to this Work are set out in our licence referred to above.

Other than as permitted in any relevant BMJ Author's Self Archiving Policies, I confirm this Work has not been accepted for publication elsewhere, is not being considered for publication elsewhere and does not duplicate material already published. I confirm all authors consent to publication of this Work and authorise the granting of this licence.

For peer review only

Broader impacts of an intervention to transform school environments: additional analyses from the INCLUSIVE cluster randomised controlled trial

1. Chris Bonell PhD[a*]
2. Matthew Dodd MSc[b]
3. Elizabeth Allen PhD[b]
4. Leonardo Bevilacqua, MSc[c]
5. Jennifer McGowan PhD[c]
6. Charles Opondo PhD[b]
7. Joanna Sturgess MSc[b]
8. Diana Elbourne PhD[b]
9. Emily Warren MSc[a]
10. Russell M. Viner PhD[c]

* Corresponding author: chris.bonell@lshtm.ac.uk; +44 (0)20 7612 7918 (no fax)

a) Department of Public Health, Environments and Society, London School of Hygiene and Tropical Medicine, 15-17 Tavistock Place, London WC1H 9SH
b) Department of Medical Statistics, London School of Hygiene and Tropical Medicine, Keppel Street, London WC1E 7HT
c) UCL Great Ormond St. Institute of Child Health, University College London, 30 Guilford St. London QC1N 1EH, UK

Words: 3,495 words

References: 26

Figure: 1

Tables: 4

For peer review only - http://bmjopen.bmj.com/site/about/guidelines.xhtml

Keywords: school; adolescent health; smoking; violence

Author contributions

All those who contributed significantly to this work are listed as authors. CB and RV directed the trial from which the data are drawn. CB, MD, EA and CO designed the analysis for this paper. MD implemented this design and undertook the analysis. DE provided additional statistical expertise. CB wrote the first draft of the paper which was then edited and commented on by MD, EA, LB, JM, CO, JS, DE, EW and RV.

Article summary

Strengths and limitations

- We report additional analyses from a rigorously conducted randomised controlled trial of a school based intervention that aimed to reduce bullying and promote health across domains (and which was found to be effective across primary and secondary outcomes).
- The new analyses are post hoc but informed by hypotheses informed by existing research and theory.

Key points

- We found evidence of multiple impacts on reduced e-cigarette use, cyber-bullying perpetration and perpetration of aggressive behaviours, as well as reduced participation in school disciplinary procedures and truancy.
- The intervention was effective in bringing about a broader range of beneficial outcomes, adding to the evidence that the intervention is a promising approach to promoting adolescent health via an intervention that is attractive to schools.

For peer review only - http://bmjopen.bmj.com/site/about/guidelines.xhtml

For peer review only

Broader impacts of an intervention to transform school environments: additional analyses from the INCLUSIVE cluster randomised controlled trial

ABSTRACT

Background

We have previously reported benefits for reduced bullying, smoking, alcohol and drug use and mental health from a trial of 'Learning Together', an intervention which aimed to modify school environments and implement restorative practice and a social and emotional skills curriculum.

Objectives

To examine additional, theory-driven analyses of broader impacts.

Design

Cluster randomized trial

Settings

40 state secondary schools in southern England

Participants

Students age 11/12 years at baseline.

Outcomes

Student self-reported measures at 24 and 36 months of: cyber-bullying victimisation and perpetration; observations of other students perpetrating aggressive behaviours at school; own perpetration of aggressive behaviours in and outside school; perceived lack of safety at school; participation in school disciplinary procedures; truancy; and e-cigarette use.

Results

For peer review only - http://bmjopen.bmj.com/site/about/guidelines.xhtml

We found evidence of multiple impacts on other health (reduced e-cigarette use, cyber-bullying perpetration, perpetration of aggressive behaviours) and educational (reduced participation in school disciplinary procedures and truancy) outcomes.

Conclusion

These analyses suggest the intervention was effective in bringing about a broader range of beneficial outcomes, adding to the evidence that the intervention is a promising approach to promoting adolescent health via an intervention that is attractive to schools.

Abtract - 187 words

Keywords: adolescent; schools; environment; trial; behaviour

For peer review only

For peer review only - http://bmjopen.bmj.com/site/about/guidelines.xhtml

Broader impacts of an intervention to transform school environments: additional analyses from the INCLUSIVE cluster randomised controlled trial

INTRODUCTION

We have previously reported the results of our main trial analyses from the INCLUSIVE cluster randomized controlled trial (RCT) of a multi-component intervention aiming to transform school environments to render these healthier places, reporting effectiveness across multiple health domains.(1) In this paper, we aim to explore the extent to which the intervention 'disrupted' the school 'system' to achieve more impacts.

There is increasing interest in the implications of 'systems' thinking for evaluating health interventions.(2) Interventions can be viewed as 'interruptions' to complex systems, the consequences of which may go beyond the primary and secondary health outcomes assessed by trials. This perspective is of particular relevance for our intervention since this aimed to promote students' health not by improving individual students' knowledge, skills or attitudes, but rather by modifying the overall school environment so that these is more engaging and thus an easier environment in which to choose healthy rather than risky behaviours. Because of our interventions' focus on settings and use of multiple components, it is particularly likely that its impact might have gone beyond our pre-hypothesised primary and secondary outcomes to impact on the school systems' broader functioning.(3) This paper therefore draws on theory to develop and test hypotheses about what the broader impacts of our intervention might have been.

Our intervention aimed to transform the school environment to make this more salutogenic, informed by previous evidence of effective interventions.(4-6) It aimed to do so by: (a) using 'restorative approaches' to address conflict; (b) rendering schools more participative by involving students and staff in an action group to review local data on student experiences and use this to change school policies relating to behaviour management, and lead the intervention; and (c) providing a social and

For peer review only - http://bmjopen.bmj.com/site/about/guidelines.xhtml

emotional skills curriculum for students age 12-15 years.(1, 7) Restorative approaches aim to enable victims to communicate to perpetrators the harms experienced, and enable perpetrators to recognise and take steps to remedy this, and avoid further harms.(8) Restorative approaches include primary prevention of conflict (via 'circle-time', which brings students together to build and maintain relationships) and/or secondary prevention to resolve incidents (such as 'conferencing' to address serious incidents).

The intervention was supported by a theory of change which was informed by an appropriate settings-based social theory: the theory of human functioning and school organisation.(9) We theorised that schools can reduce bullying and aggression by transforming the school environment to build student commitment to learning and sense of belonging in school. It was theorised that this in turn can be achieved by improving relationships between and among staff and students (via the action group and restorative practice), and by better integrating students' academic education and broader personal development (via the curriculum and restorative practice). It was further theorised that by increasing student commitment to and belonging in school, this would reduce student interest and involvement in anti-social peer groups and behaviours.

Our main trial paper reported a range of intervention effects in terms of reduced bullying victimisation and use of tobacco, alcohol and drugs, as well as improved mental wellbeing, psychological functioning and health-related quality of life among adolescent students.(1)

The first area where we anticipate beneficial broader impacts is cyber-bullying. Our main trial analyses reported effects of the intervention on our primary outcome of reduced bullying victimisation (Gatehouse Bullying Scale, GBS(10)). This is an important result given the prevalence of bullying(11) and its association with concurrent and future physical and mental health harms.(12-18) However, this analysis was insensitive to any effects of the intervention on cyber-bullying. Cyber-bullying is an increasingly prevalent aspect of bullying, associated with significant harms.(19) We did not include this in our list of primary or secondary outcomes because this mostly occurs outside school. However,

For peer review only - http://bmjopen.bmj.com/site/about/guidelines.xhtml

given that our intervention is theorised to work by decreasing student interest in anti-social behaviour in general not limited to the school site.

Our second area of exploration is perpetration of aggression. Our main trial analyses found no evidence of effects on our other primary outcome of self-reported perpetration of school-based aggression (Edinburgh Study of Youth Transitions, ESYTC, measure(20)). This was an unexpected finding given the reduction in bullying victimisation, and given the intervention was theorised to reduce bullying victimisation and perpetration of aggression via a common mechanism involving increased student commitment to school and reducing student involvement in anti-social peer groups and behaviours. A systematic review has previously concluded that trials of whole-school interventions addressing violence sometimes find effects on victimisation but not perpetration, possibly because participants under-report perpetration of socially unacceptable behaviours particularly in school.(5) Our student questionnaire also included a measure for students to report their observations of other students perpetrating aggressive behaviours, thus perhaps providing a broader assessment of aggressive behaviours in school and less prone to under-reporting. Therefore, we hypothesise that we will find effects of the intervention on this measure of student-reported observations of other students perpetrating aggressive behaviours at school. Furthermore, our primary measure of perpetration of aggression focused only on school-based behaviours. Because our intervention aimed to reduce students' general involvement in anti-social peer groups and behaviours, rather than merely reducing such behaviours in school, we hypothesise that the intervention would be effective in reducing a broader measure of students' own perpetration of aggressive behaviours not specific to school that we included in our questionnaire.

Our third focus for this paper is on impacts of the intervention on the overall functioning of the school system. Interventions effective in reducing bullying and promoting student health are more likely to be scaled up if schools and policy-makers can see evidence that such interventions also reduce school workloads and enhance education.(21) Our theory of change centred on enhancing student commitment to school and reducing student involvement in anti-social behaviours, and we found

For peer review only - http://bmjopen.bmj.com/site/about/guidelines.xhtml

effects not only for reduced bullying victimisation but also increased student commitment to school.(22) We therefore hypothesise that this will translate into students reporting: feeling safer at school; less participation in school disciplinary procedures; and less truancy.

Our final focus in this paper is on e-cigarette use. As indicated above, we found effects for smoking but our measure focused on the smoking of tobacco rather than use of e-cigarettes. However, we would also expect the intervention to reduce the latter. There are increasing concerns about the increasing prevalence of e-cigarette use among young people with some evidence that this is associated with subsequent increases in smoking tobacco.(23) We therefore hypothesise that rates of use of e-cigarettes are lower among schools in the intervention group.

In summary, we hypothesise that the intervention was effective not only in promoting the original list of intended outcomes, but also in promoting a broader range of unintended but beneficial impacts via its disruption of the school system, reducing student-reported: cyber-bullying victimisation and perpetration; observations of other students perpetrating aggressive behaviours at school; own perpetration of aggressive behaviours in and outside school; perceived lack of safety at school; participation in school disciplinary procedures; truancy; and e-cigarette use. Given, our previous finding that intervention effects on primary and secondary outcomes were apparent at 36- but not 24-month follow-up, which is in line with previous evidence that the effects of whole-school interventions build over time as they take time to transform the school environment,(6) we hypothesise that this would also apply to the outcomes examined in this paper.

METHODS

Full details of the intervention and trial are reported in full in our protocol and main trial report.(1, 7) We conducted a two-arm parallel repeat cross-sectional cluster RCT of the intervention in 40 secondary schools in south-east England. To be included, schools had government inspections rating of ‘requires improvement’ or above, and were recruited by the trial team via emails. Our student

For peer review only - http://bmjopen.bmj.com/site/about/guidelines.xhtml

population consisted of all students: at baseline in 2014 at the end of year 7 (11-12 years); at interim 24-month follow-up; and final 36-month follow-up. Students were surveyed using paper questionnaires in classes under exam conditions by trained fieldworkers blinded to allocation. After baseline surveys, schools were allocated 1:1 to intervention or control by computer-generated random numbers stratified by: single-sex versus mixed-sex school; school-level student free-school-meal eligibility (0-23%; >23%) indicating poverty; and General Certificate of Secondary Education results accounting for student baseline attainment (above/below the median score for England of 1000).

The intervention involved all staff in intervention schools receiving training to use restorative practice to prevent and address student conflicts. Approximately 5-10 key staff per school were trained in-depth to deliver restorative conferences dealing with more serious incidents. All schools received a manual to guide the convening and running of a school action group comprising at least six staff and six students, led by a member of the school's senior leadership team. An external facilitator supported action groups in the first two but not the third year of intervention, when they moved to being self-directed. Action groups reviewed anonymised finding from the school's baseline survey to understand local needs, and aimed to coordinate the intervention and revise policies so that these supported use of restorative practice. Schools were provided with materials to guide delivery of a social and emotional skills curriculum for students in years 8-10 to receive 5-10 hours teaching per year. The curriculum addressed bullying and aggression but not specific to a particular setting such as school or online. Schools in the control group continued with usual practice.

For peer review only

Our measures analysed in this paper are described in table 1. Each of these were included in student questionnaires used to assess trial outcomes but did not form part of our specified trial outcomes. We adapted Smith and colleagues' measure of cyber-bullying perpetration and victimisation.(24) We developed a new single-item measure of student-reported observations of other students perpetrating aggressive behaviours at school, where students indicated which behaviours they had observed at school to provide a quantitative measure scored 0-7. We examined students' own perpetration of aggressive behaviours in or outside school using a modified four-item version of the ESYTC measure

For peer review only - http://bmjopen.bmj.com/site/about/guidelines.xhtml

of anti-social behaviours.(20) Students reported which behaviours they had engaged in to give a quantitative score 0-4. We assessed perceived lack of school safety using a single item derived from the Healthy School Ethos (HSE) study.(25) We assessed student participation in school disciplinary procedures using the six-item ESYTC measure of school discipline.(20) This assessed students' frequency of engagement (never; one or two times; three or four times; five or more times) with six disciplinary procedures to provide a quantitative score 0-18. We assessed school truancy using a student-reported single item measure previously used in the Ripple trial.(26) We developed a new single-item measure of e-cigarette use.

PATIENT AND PUBLIC INVOLVEMENT

the trial involved young people from The National Children's Bureau Young Researchers' Group (YRG) in advising on intervention and research methods during three meetings during the set-up phase. School action groups comprised part of the intervention and enabled students to participate in planning and coordinating intervention activities.

For peer review only

TABLE 1: OUTCOME MEASURES

Outcome measure	Question		Responses	Source	Variable
Cyber-bullying victimisation	Have you been bullied through mobile phone use or on the internet in the last three months?		No I haven't<br>Yes, once or twice<br>Yes, two or three times a month<br>Yes, about once a week<br>Yes, several times a week or more	Adapted from DAPHNE measure of cyber-bullying	Binary any yes /no
Cyber-bullying perpetration	Have you ever bullied anyone else using your mobile phone or using the internet?		No I haven't<br>Yes, once or twice<br>Yes, two or three times a month<br>Yes, about once a week Yes, several times a week or more	Adapted from DAPHNE measure of cyber-bullying	Binary any yes/no
Student-reported observations of other students perpetrating aggressive behaviours at school	Which of the following have you seen happen at this school in the last 3 months of school		Boys fighting	New	Score out of 7 (point per item)
			Girls fighting		
			Someone threatening someone		
			A student trying to hurt another student		
			Someone robbing money or a mobile phone		
			Someone letting off a firework		
			Someone carrying a knife		
Perpetration of anti-social behaviour in or outside school	During the last 3 months of school	Did you ever carry a knife or other weapon with you for protection or in case it was needed in a fight?	No<br>Yes	Adapted from ESYTC measure of anti-social behaviour	Score out of 4 (4 items with each n/y 0-1)
		Did you use force, threats or a weapon to steal money or something else from somebody?			
		Did you damage or destroy property that did not belong to you on purpose			

For peer review only

		(e.g. windows, cars or street lights)?			
		Did you ever set fire or try to set fire to something on purpose (e.g. bus shelter, shop, etc.)?			
Participation in school disciplinary procedures	During the last 3 months of school how often did these things happen to you because of something you had done wrong?	The school got in touch with my parents by letter or telephone about an incident	0 times<br>1 or 2 times<br>3 or 4 times<br>5 or more times	ESYTC measure of school discipline	Score out of 18 (6 items with each scored 0-3)
		I got a punishment and my parents were informed about that			
		I was given detention			
		I was sent to the head of year, deputy head or head teacher for my behaviour			
		I was put on a conduct/behaviour sheet			
		I was given extra homework to do			
Truancy	During the last 3 months of school have you skipped / bunked off school?		No<br>Yes	Ripple measure of truancy	Binary y/n
E-cigarette use	Which of the following best describes you?		I currently smoke e cigarettes<br>I have tried e cigarettes in the past 12 months but do not currently smoke them	New	Binary – ever/ never

For peer review only

For peer review only - http://bmjopen.bmj.com/site/about/guidelines.xhtml

		I have tried e cigarettes longer than 12 months ago but do not currently smoke them<br>I have never tried e-cigarettes		
Perceived lack of school safety	Do you feel safe at this school?	All of the time<br>Most of the time Some of the time<br>Never	HSE measure of school safety	Binary – some of the time/never versus other options

For peer review only

As with our analysis of primary and secondary outcomes, our analyses of outcomes in this paper were intention-to-treat, including all schools and participants at each wave. Each outcome measure was analysed using a separate mixed-model with the measure from each time point treated as a repeated measures. Fixed effects of arm (intervention versus control), time (baseline, 24 months and 36 months), and the interaction between arm and time were specified, and estimated baseline measures were constrained to be identical in the two arms of the trial. This is equivalent to adjusting for baseline but enables data from all participants to contribute to the analysis, even where there are missing data at follow up. We specified random effects for school and participants, to allow for correlations within schools and repeated measures within participants. We undertook analyses adjusted for baseline measures of outcomes, sex, ethnicity, socioeconomic status (Family Affluence Scale, FAS) as well as for the school stratifying factors.

We used appropriate multilevel models to examine the effects of the intervention. For quantitative measures, we used mixed linear-regression models with random effects at the level of participants and schools to estimate adjusted mean differences between arms. For binary outcomes, we report unadjusted and adjusted odds ratios. Evidence for any moderation of intervention effects on our outcomes by student sex and socioeconomic status (Family Affluence Scale) was assessed by Wald tests for the treatment by subgroup interaction terms. We also calculated Cronbach's alphas to assess the inter-item reliability of our multi-question measures of student perpetration of aggressive behaviours in or outside school and student participation in school disciplinary procedures.

The trial was prospectively registered as ISRCTN10751359 with the ISRCTN Registry on 30/01/2014, and approved by the UCL Ethics Committee (ref 5248/001). Informed consent was sought from head-teachers for randomisation and intervention, and from students, deemed competent by schools to do so, for participation in surveys. Parents were informed about the study and could withdraw their children from research activities.

For peer review only

RESULTS

In total, 6,667 students in the 40 schools provided data at baseline, representing a participation rate of 93.6% of registered students (92.9% in intervention arm; 94.3% control arm). Student characteristics and baseline reports of the outcomes examined in this paper are reported in table 2, with good balance between arms.

TABLE 2: CHARACTERISTICS OF SCHOOLS AND STUDENTS AT BASELINE BY TRIAL ARM

**School characteristics**	**Control 20 schools**	**Intervention 20 schools**	**Overall 40 schools**
School sex mix, n (%)			
Mixed	15 (75·0)	15 (75·0)	30 (75·0)
Girls	3 (15·0)	4 (20·0)	7 (17·5)
Boys	2 (10·0)	1 (5·0)	3 (7·5)
Ofsted rating*, n (%)			
Excellent	5 (25·0)	6 (30·0)	11 (27·5)
Good	13 (65·0)	12 (60·0)	25 (62·5)
Requires improvement	2 (10·0)	2 (10·0)	4 (10·0)
Value added score, mean (SD)	1,003 (24·8)	1,004 (20·4)	1,003 (22·4)
Proportion of students on free school means, mean (SD)	36 (18·0)	35 (22·0)	36 (20·0)
Income Deprivation Affecting Children Index (IDACI), mean (SD)	0·26 (0·2)	0·24 (0·2)	0·25 (0·2)
**Student socio-demographic characteristics**	**3,347 students†**	**3,320 students†**	**6,667 students†**
Age, mean (SD)	12 (0·4)	12 (0·4)	12 (0·4)
Sex, n (%)			
Male	1,639 (49·9)	1,464 (44·9)	3,103 (47·3)
Female	1,649 (50·2)	1,804 (55·2)	3,453 (52·7)
Ethnicity, n (%)			
White British	1,391 (41·5)	1,221 (37·3)	2,612 (39·7)
White other	291 (8·8)	273 (8·3)	564 (8·6)
Asian/Asian British	859 (25·9)	786 (24·0)	1,645 (25·0)
Black/Black British	384 (11·6)	535 (16·4)	919 (14·0)
Chinese/Chinese British	11 (0·3)	35 (1·1)	46 (0·7)
Mixed ethnicity	238 (7·2)	224 (6·9)	462 (7·0)
Other	140 (4·2)	198 (6·1)	338 (5·1)
Family affluence scale, mean (SD)	6 (1·8)	6 (1·8)	6 (1·8)
**Student baseline rates of outcomes**			
Cyber-bullying perpetration, n (%)	290 (8·9)	279 (8·6)	569 (8·7)
Cyber-bullying victimisation, n (%)	522 (16·0)	467 (14·5)	989 (15·3)
Truancy, n (%)	189 (5·9)	182 (5·8)	371 (5·8)
E-cigarette use, n (%)	187 (5·8)	131 (4·2)	318 (5·0)
Perceived lack of school safety, n (%)	493 (15·6)	440 (14·5)	933 (15·1)
Student-reported observations of other students perpetrating aggressive behaviours at school, mean (SD)	2·30 (1·61)	2·04 (1·65)	2·17 (1·64)
Perpetration of aggressive behaviour in/outside school, mean (SD)	0·06 (0·31)	0·06 (0·33)	0·06 (0·32)

For peer review only - http://bmjopen.bmj.com/site/about/guidelines.xhtml

School characteristics	Control	Intervention	Overall
Participation in school disciplinary procedures, mean (SD)	2·47 (2·96)	2·39 (3·00)	2·43 (2·98)

* One control school did not have an Ofsted rating
† The number of students who responded at this survey; actual number of responses to each question varies, but item non-response is similar across arms.

All schools participated in the surveys at 24 and 36 months, with student participation rates being similar by arms (figure 1).

Cronbach's alphas for our ESYTC measures of student perpetration of aggressive behaviours in or outside school was 0.55 and for our ESYTC measure of student participation in school disciplinary procedures was 0.79.

Our broader student outcomes at 24 and 36 months are reported in table 3. At 24 months comparing intervention with control schools, we found lower rates of: cyber-bullying victimisation (P=0.035) but not perpetration; and e-cigarette use (P=0.002). Students in intervention schools were more likely to report lack of perceived school safety at 24 months than controls (P=0.006). There was no evidence of difference between arms in: student-reported observations of other students perpetrating aggressive behaviours at school; perpetration of aggressive behaviours in or outside school; or truancy. There was lower reported participation in school disciplinary procedures in intervention compared with control schools at 24 months, of borderline statistical significance (P=0.043).

For peer review only

TABLE 3: OUTCOMES AT 24- AND 36-MONTH FOLLOW-UP

Outcomes	24 months			36 months		
	Control n/N (%) or mean (SD), N (%)	Intervention n/N (%) or mean (SD), N (%)	Adjusted OR or MD (95% confidence interval), P-value	Control n/N (%) or mean (SD), N (%)	Intervention n/N (%)	Adjusted OR or MD (95% confidence interval), P-value
Cyber-bullying victimisation	443/3116 (14.2%)	340/2993 (11.4%)	OR= 0.77 (0.61 to 0.98), 0.035	347/2987 (11.6%)	266/2754 (9.7%)	OR=0.80 (0.62 to 1.05), 0.11
Cyber-bullying perpetration	257/3112 (8.3%)	229/2984	OR=0.90 (0.67 to 1.19), 0.45	287/3008 (9.5%)	193/2766 (7.0%)	OR=0.65 (0.48 to 0.88), 0.005

Perceived lack of school safety	543/3106 (17.5%)	568/2944 (7.7%)	OR=1.39 (1.10 to 1.75), 0.006	601/2919 (20.6%)	532/2682 (19.8%)	OR=1.05 (0.83 to 1.34), 0.44
Student-reported observations of other students perpetrating aggressive behaviours at school	2.43 (1.65), 3179	2.12 (1.71), 3063	MD= -0.08 (-0.18 to 0,01), 0.096	2.23 (1.77), 3069	2.07 (1.76), 2834	MD=0.10 (0 to 0.20), 0.049
Perpetration of aggressive behaviours in/ outside school	0.10 (0.44), 3149	0.08 (0.39), 3017	MD=-0.009 (-0.034 to 0.015), 0.46	0.12 (0.50), 3023	0.09 (0.43), 2778	MD=-0.031 (-0.056 to -0.006), 0.016
Participation in school disciplinary procedures	2.74 (3.28), 3128	2.36 (3.12), 3009	MD=-0.16 (-0.32 to 0), 0.043	2.55 (3.21), 3017	2.15 (2.94), 2757	MD=-0.32 (-0.48 to -0.15), <0.001
Truancy	340/3088 (11.0%)	299/2961 (10.1%)	OR=0.92 (0.70 to 1.21), 0.55	426/2981 (14.3%)	294/2750 (10.7%)	0.64 (0.49 to 0.84), 0.001
E-cigarette use	558/3061 (18.2%)	352/2913 (12.1%)	OR=0.60 (0.43 to 0.83), 0.002	630/2946 (21.4%)	394/2692 (14.6%)	0.59 (0.42 to 0.82), 0.002

For peer review only

At 36 months comparing intervention and control schools, we found reduced rates of: cyber-bullying perpetration (P=0.005) but not victimisation; perpetration of aggression in or outside school (P=0.016); participation in school disciplinary procedures (P<0.001); truancy (P=0.001); and e-cigarette use (P=0.002). There was lower student-reported observations of other students perpetrating aggressive behaviours at school, of borderline significance (P=0.049). There were no evidence of difference in perceived school safety.

Table 4 presents outcomes and follow-up points for which there was evidence of moderation. We found evidence that intervention effects on cyber-bullying perpetration at 24 months were moderated by student sex, such that effects were larger for boys (P=0.002). Intervention effects on observed aggression by other students at 24 months were moderated by student sex and socioeconomic status, with effects larger for girls (P=0.02) and affluent students (P=0.03). Effects on perceived lack of school safety at 24 months were larger for girls (P=0.001), and at 36 months were larger for students from poorer families (P=0.002). Effects on participations in school disciplinary procedures at 24 and 36 months were larger for boys (P<0.001; P=0.001 respectively). Effects on truancy at 24 months

For peer review only - http://bmjopen.bmj.com/site/about/guidelines.xhtml

were larger for boys (P=0.015). Effects on e-cigarette use at 24 and 36 months were larger for boys (P=0.014; P<0.001 respectively).

TABLE 4: MODERATION

**Outcome**	**Follow-up (months) where evidence of moderation**	**Moderator**		**Association (95% CI)**	**P value**
		Variable	***Categories***		
Perpetration cyber-bullying	24	Sex	Boys	0.61 (0.41, 0.89)	0.002
			Girls	1.19 (0.85, 1.67)	
Observed aggression	24	Sex	Boys	0.01 (-0.11, 0.13)	0.02
			Girls	-0.15 (-0.26, -0.04)	
		Family affluence	Low	-0.18 (-0.62, 0.25)	0.03
			Middle	0.04 (-0.09, 0.18)	
			High	-0.14 (-0.25, -0.03)	
Lack of safety	24	Sex	Boys	0.99 (0.73, 1.35)	0.001
			Girls	1.74 (1.33, 2.27)	
	36	Family affluence	Low	3.07 (0.99-9.54)	0.002
			Middle	0.72 (0.51, 1.01)	
			High	1.23 (0.94, 1.60)	
Participation in school disciplinary procedures	24	Sex	Boys	-0.39 (-0.60, -0.19)	<0.001
			Girls	0.02 (-0.17, 0.20)	
	36	Sex	Boys	-0.55 (-0.76, -0.34)	0.001
			Girls	-0.14 (-0.33, 0.05)	
Truancy	24	Sex	Boys	0.69 (0.48, 0.99)	0.015
			Girls	1.13 (0.83-1.55)	
E-cigarette use	24	Sex	Boys	0.45 (0.30, 0.67)	0.014
			Girls	0.80 (0.54, 1.20	
	36		Boys	0.35 (0.23, 0.53	<0.001
			Girls	0.94 (0.63, 1.40).	

For peer review only

DISCUSSION

Summary of key findings

We report an analysis of broader system impacts on student health and school functioning outcomes of this settings-based intervention aim to render schools more health-promoting environments, which was previously reported to be effective in reducing bullying victimisation and use of alcohol, tobacco and drugs, as well as promoting mental and physical health.(1) We found evidence at 36 but not 24 months of intervention effects on: cyber-bullying perpetration; student observations of aggression by other students; students' own perpetration of aggressive behaviours in or outside school; truancy; and participation in school disciplinary procedures. There was evidence of an effect on increased student perceptions of lack of school safety at 24 but not 36 months. There was also evidence of an effect at 24 but not 36 months on cyber-bullying victimisation. We found evidence of an effect on e-cigarette use at both time-points.

These findings suggest that our intervention, which aimed to reduce bullying via making schools more engaging environments, and which did not explicitly focus on cyber-bullying, might nonetheless have been effective in reducing this. Our findings also suggest that the intervention might, contrary to the main analyses, have reduced rates of aggression including aggression beyond the school environment. However, results across time-points and measures are somewhat inconsistent, probably as a result of chance. Our intervention also appears to have reduced student use of e-cigarettes, which is an important finding given increasing concerns about this as a gateway to tobacco use.(23) This evidence of additional health impacts provide further evidence in support of the intervention theory of change, that it is possible to improve young people's health across a range of areas by addressing the school as

For peer review only - http://bmjopen.bmj.com/site/about/guidelines.xhtml

a potentially salutogenic environment rather than merely as a setting for individual-focused health education in classrooms. Furthermore, our finding of broader impacts on school functioning in terms of reduced truancy and student involvement in discipline systems, provides evidence of the knock-on consequences of a health intervention disrupting the school system to achieve impacts in the domain of education.

Our finding of increased student perceptions of lack of safety in intervention versus control schools at 24 months is of concern, although this was not found at 36 months. We note that this association runs counter to our previous findings for actual rates of bullying victimisation(1) and to the findings in this paper on aggression. This finding may be due to chance. However, it may might be explained by the intervention's focus on bullying and aggression sensitizing students to issues of safety, leading them to feel unsafe at the initial (24 month) follow-up but with this dissipating as the intervention became normalised and exerted positive effects on bullying and aggression.

Intervention effects were moderated in some cases by student sex and family affluence. Effects were larger for boys regarding reduced cyber-bullying perpetration and truancy at 24 months, and reduced participations in school disciplinary procedures and e-cigarette use at 24 and 36. For girls, intervention effects were greater regarding reduced observed aggression by other students and decreased perceived school safety at 24 months. Effects for decreased perceived school safety at 36 months were also larger for students from poorer families. Effects on reduced observed aggression at 24 months were larger for affluent students.

These moderator analyses add to the evidence from our main analyses that the intervention might generally have been more effective for boys than girls. As with our main trial analyses, there was less evidence for moderation by socioeconomic status. These findings of gender inequity of effects is in line with some previous research suggesting that whole-school interventions, including those to reduce violence, can sometimes be more effective for boys than girls.(1, 4) This might because, in

For peer review only - http://bmjopen.bmj.com/site/about/guidelines.xhtml

such interventions, violence and other problem behaviours among boys receive more attention than those experienced by girls.

Strengths and limitations

The study was a rigorously conducted experimental evaluation. These are additional analyses not included in our original protocol. Therefore, caution is required in the interpretation of significant findings. However, the analyses are guided by explicit, theory-driven hypotheses, as set out in our introduction, rather than being the product of subjecting all measures to analysis and merely reporting significant findings. We relied on student self-reports as these were less likely than school routine data to be biased by delivery of the intervention. Where possible, we used reliable existing measures. While our measure of student participation in disciplinary procedures had high inter-item reliability, this was lower for our measure of student perpetration of aggressive behaviours in or outside school so our conclusions regarding effects on this outcome should be cautious.

Implications for policy and research

Our findings suggest that the intervention disrupted school systems to achieve a range of unintended but beneficial impacts on student health and school functioning not captured in the main trial analyses.(2, 3) These results suggest that it is possible to achieve public health improvements across a range of outcomes using a single coordinated intervention which focuses on environmental transformation rather than individual behaviour-change. This is important given the impracticality of implementing different interventions for multiple outcomes in schools.(21) Our findings also suggest important benefits for education and school functioning. It appears that the intervention's previously reported effects on reducing bullying victimisation and improving student commitment to school(1, 22) translated into reduced student truancy and participation in school disciplinary procedures. This is especially important evidence for school leaders suggesting the potential educational benefits of

For peer review only - http://bmjopen.bmj.com/site/about/guidelines.xhtml

whole-school health interventions. Our analyses suggest this intervention worked more effectively for boys than girls. Further research is needed into how to ensure school-based interventions are more equitable, perhaps by ensuring these address less overt forms of student disengagement and conflict.

ACKNOWLEDGEMENTS

We are grateful to the staff and students of participating schools for their dedication to the intervention and completion of the outcome surveys and process evaluation surveys and interviews. We are very grateful for the advice and support of our Trial Steering Committee and Data Monitoring Committee. All those who contributed significantly to this work are listed in these acknowledgements.

COMPETING INTERESTS

None to declare.

This article has not been submitted and will not be submitted for publication elsewhere while under consideration by BMJ Open. The paper includes data (on response rates and baseline comparisons between intervention and control groups) that was included in the paper cited below. Other analyses are new to this paper.

Bonell C, Allen E, Warren E, McGowan J, Bevilacqua L, Jamal F, et al. Initiating change in the school environment to reduce bullying and aggression: a cluster randomised controlled trial of the Learning Together (LT) intervention in English secondary schools. The Lancet. 2018;392(10163):2452-64.

For peer review only

FUNDING

This work was supported by the National Institute for Health Research (NIHR) in England under its Public Health Research Board (12/153/60) and the Education Endowment Foundation (no grant number). The views expressed in this publication are those of the authors and do not necessarily reflect those of the National Health Service (NHS), the NIHR or the Department of Health for England. The study funders (National Institute for Health Research and Education Endowment Foundation played no role in the: study; collection, analysis and interpretation of data; the writing of the report; or the decision to submit the manuscript for publication.

DATA STATEMENT

Anonymised data from the study are available on request.

For peer review only

REFERENCES

1. Bonell C, Allen E, Warren E, McGowan J, Bevilacqua L, Jamal F, et al. Initiating change in the school environment to reduce bullying and aggression: a cluster randomised controlled trial of the Learning Together (LT) intervention in English secondary schools. The Lancet. 2018;392(10163):2452-64.
2. Moore GF, Evans RE, Hawkins J, Littlecott H, Melendez-Torres GJ, Bonell GJ, et al. From complex social interventions to interventions in complex social systems: future directions and unresolved questions for intervention development and evaluation. Evaluation and Program Planning. 2019;;25(1):23-45.
3. Rosas SR. Systems thinking and complexity: considerations for health promoting schools Health Promotion International. 2017;32(2):301-11.
4. Flay BR, Graumlich S, Segawa E, Burns JL, Holliday MY. Effects of 2 prevention programs on high-risk behaviors among African American youth: a randomized trial. Archives of Pediatrics & Adolescent Medicine. 2004;158(4):377-84.
5. Langford R, Bonell CP, Jones HE, Pouliou T, Murphy SM, Waters E, et al. The WHO Health Promoting School framework for improving the health and well-being of students and staff. Cochrane Database of Systematic Reviews 2011, Issue 1 Art No: CD008958. 2014.
6. Patton G, Bond L, Carlin JB, Thomas L, Butler H, Glover S, et al. Promoting social inclusion in schools: group-randomized trial of effects on student health risk behaviour and well-being. American Journal of Public Health. 2006;96(9):1582-7.
7. Bonell C, Allen E, Christie D, Elbourne D, Fletcher A, Grieve R, et al. Initiating change locally in bullying and aggression through the school environment (INCLUSIVE): study protocol for a cluster randomised controlled trial. Trials. 2014;15:381.
8. Morrison B. Restorative justice in schools. In: Elliot E, Gordon RM, editors. New directions in restorative justice: Issues, practices, evaluation. Cullompton, UK: Willan Publishing; 2005. p. 26-52.
9. Markham WA, Aveyard P. A new theory of health promoting schools based on human functioning, school organisation and pedagogic practice. Social Science & Medicine. 2003;56(6):1209-20.
10. Bond L, Wolfe S, Tollit M, Butler H, Patton G. A comparison of the Gatehouse Bullying Scale and the peer relations questionnaire for students in secondary school. Journal of School Health. 2007;77(2):75-9.
11. Inchley J, Currie D, Young T, Samdal O, Torsheim T, Augustson L, et al. Growing up unequal: gender and socioeconomic differences in young people's health and well-being. Copenhagen: WHO Europe, 2016 Contract No.: ISBN 978 92 890 5136 1.
12. Arseneault L. Annual Research Review: The persistent and pervasive impact of being bullied in childhood and adolescence: implications for policy and practice. J Child Psychol Psychiatry. 2017.
13. Hair EC, Park MJ, Ling TJ, Moore KA. Risky behaviors in late adolescence: co-occurrence, predictors, and consequences. J Adolesc Health. 2009;45(3):253-61.
14. Copeland WE, Wolke D, Angold A, Costello EJ. Adult psychiatric outcomes of bullying and being bullied by peers in childhood and adolescence. JAMA Psychiatry. 2013;70(4):419-26.
15. Lereya ST, Copeland WE, Costello EJ, Wolke D. Adult mental health consequences of peer bullying and maltreatment in childhood: two cohorts in two countries. Lancet Psychiatry. 2015;2(6):524-31.
16. Fisher HL, Moffitt TE, Houts RM, Belsky DW, Arseneault L, Caspi A. Bullying victimisation and risk of self harm in early adolescence: longitudinal cohort study. BMJ. 2012;344:e2683.
17. Winsper C, Lereya T, Zanarini M, Wolke D. Involvement in bullying and suicide-related behavior at 11 years: a prospective birth cohort study. J Am Acad Child Adolesc Psychiatry. 2012;51(3):271-82 e3.
18. Fantaguzzi C, Allen E, Miners A, Christie D, Opondo C, Sadique Z, et al. Health-related quality of life associated with bullying and aggression: a cross-sectional study in English secondary schools. Eur J Health Econ. 2017.
19. Bevilacqua L, Shackleton N, Hale D, Allen E, Bond L, Christie D, et al. The role of family and school-level factors in bullying and cyberbullying: a cross-sectional study. BMC Pediatr. 2017;17(1):160.
20. Smith DJ. School experience and delinquency at ages 13 to 16. Edinburgh: Centre for Law and Society, University of Edinburgh; 2006.
21. Bonell C, Humphrey N, Fletcher A, Moore L, Anderson R, Campbell R. Why schools should promote students' health and wellbeing Education policy shouldn't focus solely on academic attainment. British Medical Journal. 2014;348:g3078.
22. Bonell C, Allen E, Opondo C, Warren E, Elbourne D, Sturgess J, et al. Examining intervention mechanisms of action using mediation analysis within a randomised trial of a whole-school health intervention. Journal of Epidemiology and Community Health http://dxdoiorg/101136/jech-2018-211443. 2019.

For peer review only - http://bmjopen.bmj.com/site/about/guidelines.xhtml

23. Barrington-Trimis JL, Kong G, Leventhal AM, Liu F, Mayer M, Boley Cruz T, et al. E-cigarette use and subsequent smoking frequency among adolescents. Pediatrics. 2018;142(6):e20180486.
24. Smith PK, Mahdavi J, Carvalho M, Fisher S, Russell S, Tippett N. Cyberbullying: Its nature and impact in secondary school pupils. Journal of Child Psychology and Psychiatry. 2008;49(376):e385.
25. Bonell C, Sorhaindo A, Allen E, Strange V, Wiggins M, Fletcher A, et al. Pilot multi-method trial of a school-ethos intervention to reduce substance use: building hypotheses about upstream pathways to prevention. Journal of Adolescent Health. 2010;47(6):555-63.
26. Stephenson JM, Strange V, Forrest S, Oakley A, Copas A, Allen E, et al. Pupil-led sex education in England (RIPPLE study): cluster-randomised intervention trial. Lancet. 2004;364(9431):338-46.

For peer review only

For peer review only - http://bmjopen.bmj.com/site/about/guidelines.xhtml

FIGURE 1: TRIAL PARTICIPANTS

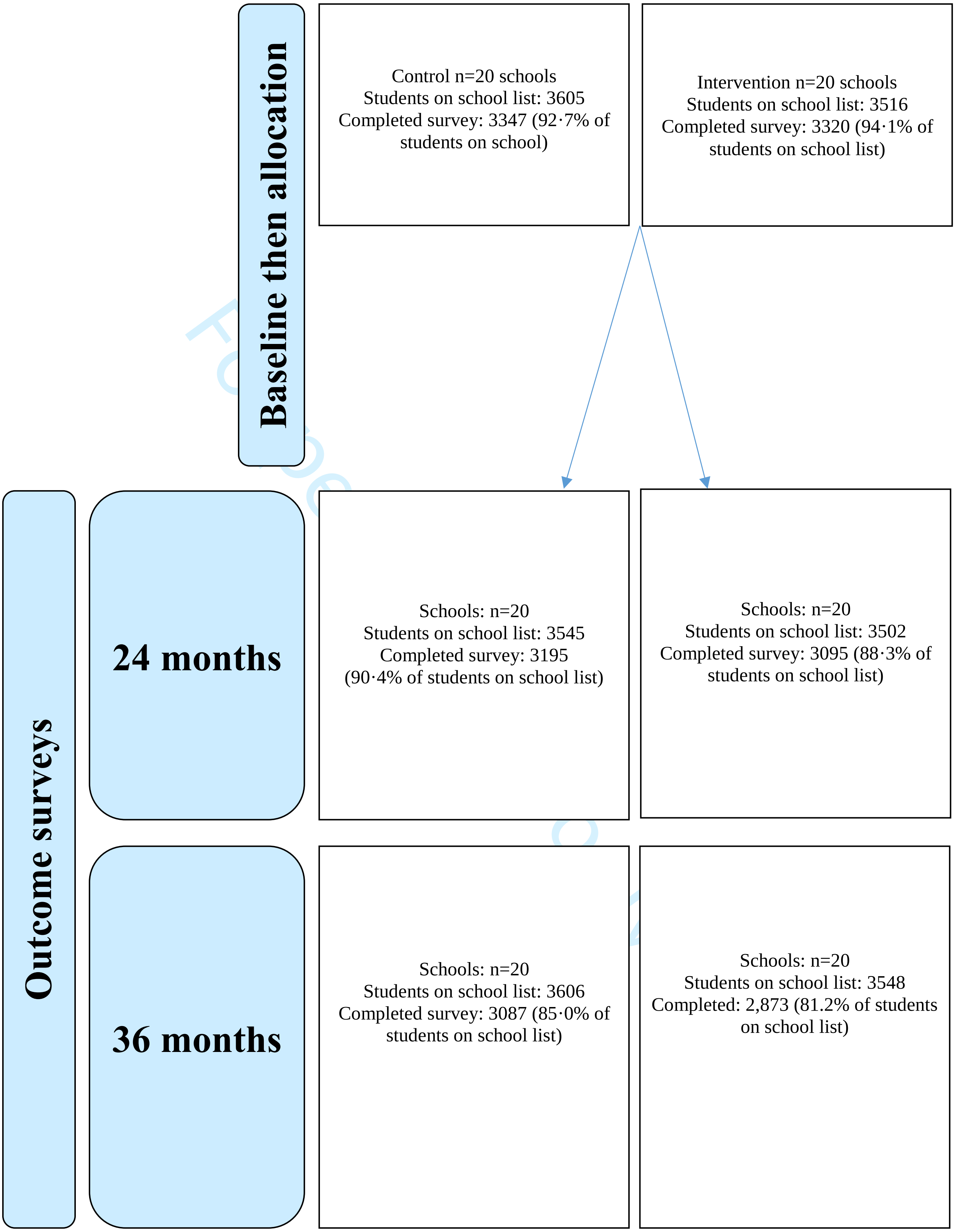

CONSORT 2010 checklist of information to include when reporting a randomised trial*

Section/Topic	Item No	Checklist item	Reported on page No
**Title and abstract**			
	1a	Identification as a randomised trial in the title	1,3,5
	1b	Structured summary of trial design, methods, results, and conclusions (for specific guidance see CONSORT for abstracts)	3,4
**Introduction**			
Background and objectives	2a	Scientific background and explanation of rationale	5-8
	2b	Specific objectives or hypotheses	8
**Methods**			
Trial design	3a	Description of trial design (such as parallel, factorial) including allocation ratio	8
	3b	Important changes to methods after trial commencement (such as eligibility criteria), with reasons	NA
Participants	4a	Eligibility criteria for participants	8,9
	4b	Settings and locations where the data were collected	9
Interventions	5	The interventions for each group with sufficient details to allow replication, including how and when they were actually administered	9
Outcomes	6a	Completely defined pre-specified primary and secondary outcome measures, including how and when they were assessed	Additional analysis. 9-13
	6b	Any changes to trial outcomes after the trial commenced, with reasons	NA
Sample size	7a	How sample size was determined	reference to main trial report
	7b	When applicable, explanation of any interim analyses and stopping guidelines	NA
Randomisation:			9 + reference to main trial report
Sequence generation	8a	Method used to generate the random allocation sequence	
	8b	Type of randomisation; details of any restriction (such as blocking and block size)	9
Allocation concealment	9	Mechanism used to implement the random allocation sequence (such as sequentially numbered containers), describing any steps taken to conceal the sequence until interventions were assigned	9 + reference to main trial

For peer review only - http://bmjopen.bmj.com/site/about/guidelines.xhtml

mechanism			report
Implementation	10	Who generated the random allocation sequence, who enrolled participants, and who assigned participants to interventions	9 + reference to main trial report
Blinding	11a	If done, who was blinded after assignment to interventions (for example, participants, care providers, those assessing outcomes) and how	9
	11b	If relevant, description of the similarity of interventions	NA
Statistical methods	12a	Statistical methods used to compare groups for primary and secondary outcomes	14 + reference to main trial report
	12b	Methods for additional analyses, such as subgroup analyses and adjusted analyses	14
**Results**			
Participant flow (a diagram is strongly recommended)	13a	For each group, the numbers of participants who were randomly assigned, received intended treatment, and were analysed for the primary outcome	15, figure 1 + reference to main trial report
	13b	For each group, losses and exclusions after randomisation, together with reasons	15, 16 + reference to main trial report
Recruitment	14a	Dates defining the periods of recruitment and follow-up	9
	14b	Why the trial ended or was stopped	NA
Baseline data	15	A table showing baseline demographic and clinical characteristics for each group	Table 2
Numbers analysed	16	For each group, number of participants (denominator) included in each analysis and whether the analysis was by original assigned groups	Tables 3-4
Outcomes and estimation	17a	For each primary and secondary outcome, results for each group, and the estimated effect size and its precision (such as 95% confidence interval)	Table 3
	17b	For binary outcomes, presentation of both absolute and relative effect sizes is recommended	reference to main trial report
Ancillary analyses	18	Results of any other analyses performed, including subgroup analyses and adjusted analyses, distinguishing pre-specified from exploratory	Table 3

For peer review only - http://bmjopen.bmj.com/site/about/guidelines.xhtml
For peer review only

Harms	19	All important harms or unintended effects in each group (for specific guidance see CONSORT for harms)	reference to main trial report
**Discussion**			
Limitations	20	Trial limitations, addressing sources of potential bias, imprecision, and, if relevant, multiplicity of analyses	23
Generalisability	21	Generalisability (external validity, applicability) of the trial findings	23
Interpretation	22	Interpretation consistent with results, balancing benefits and harms, and considering other relevant evidence	23
**Other information**			
Registration	23	Registration number and name of trial registry	14
Protocol	24	Where the full trial protocol can be accessed, if available	14
Funding	25	Sources of funding and other support (such as supply of drugs), role of funders	24-25

*We strongly recommend reading this statement in conjunction with the CONSORT 2010 Explanation and Elaboration for important clarifications on all the items. If relevant, we also recommend reading CONSORT extensions for cluster randomised trials, non-inferiority and equivalence trials, non-pharmacological treatments, herbal interventions, and pragmatic trials. Additional extensions are forthcoming: for those and for up to date references relevant to this checklist, see www.consort-statement.org.

For peer review only

For peer review only - http://bmjopen.bmj.com/site/about/guidelines.xhtml

BMJ Open

Broader impacts of an intervention to transform school environments on student behaviour and school functioning: additional analyses from the INCLUSIVE cluster randomised controlled trial

Journal:	*BMJ Open*
Manuscript ID	bmjopen-2019-031589.R1
Article Type:	Original research
Date Submitted by the Author:	06-Nov-2019
Complete List of Authors:	Bonell, Christopher; London School of Hygiene and Tropical Medicine, Public Health and Policy<br>Dodd, Matthew; London School of Hygiene and Tropical Medicine, Public Health and Policy<br>Allen, Elizabeth; London School of Hygiene & Tropical Medicine, Department of Medical Statistics<br>Bevilacqua, Leonardo; University College London<br>McGowan, Jennifer; University College London<br>Opondo, Charles; London School of Hygiene and Tropical Medicine<br>Sturgess, Joanna; London School of Hygiene and Tropical Medicine, Department of Population Health<br>Elbourne, Diana; LSHTM, EPH<br>Warren, Emily; London School of Hygiene & Tropical Medicine, Faculty of Epidemiology and Population Health<br>Viner, Russell; UCL Institute of child health, Population, policy and practice research programme
**Primary Subject Heading**:	Public health
Secondary Subject Heading:	Epidemiology, Smoking and tobacco
Keywords:	PUBLIC HEALTH, EPIDEMIOLOGY, STATISTICS & RESEARCH METHODS

SCHOLARONE™
Manuscripts

BMJ

I, the Submitting Author has the right to grant and does grant on behalf of all authors of the Work (as defined in the below author licence), an exclusive licence and/or a non-exclusive licence for contributions from authors who are: i) UK Crown employees; ii) where BMJ has agreed a CC-BY licence shall apply, and/or iii) in accordance with the terms applicable for US Federal Government officers or employees acting as part of their official duties; on a worldwide, perpetual, irrevocable, royalty-free basis to BMJ Publishing Group Ltd ("BMJ") its licensees and where the relevant Journal is co-owned by BMJ to the co-owners of the Journal, to publish the Work in this journal and any other BMJ products and to exploit all rights, as set out in our licence.

The Submitting Author accepts and understands that any supply made under these terms is made by BMJ to the Submitting Author unless you are acting as an employee on behalf of your employer or a postgraduate student of an affiliated institution which is paying any applicable article publishing charge ("APC") for Open Access articles. Where the Submitting Author wishes to make the Work available on an Open Access basis (and intends to pay the relevant APC), the terms of reuse of such Open Access shall be governed by a Creative Commons licence – details of these licences and which Creative Commons licence will apply to this Work are set out in our licence referred to above.

Other than as permitted in any relevant BMJ Author's Self Archiving Policies, I confirm this Work has not been accepted for publication elsewhere, is not being considered for publication elsewhere and does not duplicate material already published. I confirm all authors consent to publication of this Work and authorise the granting of this licence.

For peer review only

Broader impacts of an intervention to transform school environments on student behaviour and school functioning: additional analyses from the INCLUSIVE cluster randomised controlled trial

1. Chris Bonell PhD[a*]
2. Matthew Dodd MSc[b]
3. Elizabeth Allen PhD[b]
4. Leonardo Bevilacqua, MSc[c]
5. Jennifer McGowan PhD[c]
6. Charles Opondo PhD[b]
7. Joanna Sturgess MSc[b]
8. Diana Elbourne PhD[b]
9. Emily Warren MSc[a]
10. Russell M. Viner PhD[c]

* Corresponding author: chris.bonell@lshtm.ac.uk; +44 (0)20 7612 7918 (no fax)

a) Department of Public Health, Environments and Society, London School of Hygiene and Tropical Medicine, 15-17 Tavistock Place, London WC1H 9SH
b) Department of Medical Statistics, London School of Hygiene and Tropical Medicine, Keppel Street, London WC1E 7HT
c) UCL Great Ormond St. Institute of Child Health, University College London, 30 Guilford St. London QC1N 1EH, UK

Words: 3,495 words

References: 26

Figure: 1

Tables: 4

For peer review only - http://bmjopen.bmj.com/site/about/guidelines.xhtml

Keywords: school; adolescent health; smoking; violence

Author contributions

All those who contributed significantly to this work are listed as authors. CB and RV directed the trial from which the data are drawn. CB, MD, EA and CO designed the analysis for this paper. MD implemented this design and undertook the analysis. DE provided additional statistical expertise. CB wrote the first draft of the paper which was then edited and commented on by MD, EA, LB, JM, CO, JS, DE, EW and RV.

Article summary

Strengths and limitations

-
- The study was a rigorously conducted experimental evaluation.
- These are additional analyses not included in our original protocol so caution is required in the interpretation of significant findings.
- However, the analyses are guided by explicit, theory-driven hypotheses, as set out in our introduction, rather than being the product of subjecting all measures to analysis and merely reporting significant findings.
- We relied on student self-reports as these were less likely than school routine data to be biased by delivery of the intervention.
- While our measure of student participation in disciplinary procedures had high inter-item reliability, this was lower for our measure of student perpetration of aggressive behaviours in or outside school so our conclusions regarding effects on this outcome should be cautious.
-

For peer review only

For peer review only

For peer review only - http://bmjopen.bmj.com/site/about/guidelines.xhtml

Broader impacts of an intervention to transform school environments on student behaviour and school functioning: additional analyses from the INCLUSIVE cluster randomised controlled trial

ABSTRACT

Background

We have previously reported benefits for reduced bullying, smoking, alcohol and other drug use and mental health from a trial of ‘Learning Together’, an intervention which aimed to modify school environments and implement restorative practice and a social and emotional skills curriculum.

Objectives

To examine additional, theory-driven analyses of broader impacts.

Design

Cluster randomized trial

Settings

40 state secondary schools in southern England

Participants

Students age 11/12 years at baseline.

Outcomes

Student self-reported measures at 24 and 36 months of: cyber-bullying victimisation and perpetration; observations of other students perpetrating aggressive behaviours at school; own perpetration of aggressive behaviours in and outside school; perceived lack of safety at school; participation in school disciplinary procedures; truancy; and e-cigarette use.

Results

For peer review only

We found evidence of multiple impacts on other health (reduced e-cigarette use, cyber-bullying perpetration, perpetration of aggressive behaviours) and educational (reduced participation in school disciplinary procedures and truancy) outcomes.

Conclusion

These analyses suggest the intervention was effective in bringing about a broader range of beneficial outcomes, adding to the evidence that the intervention is a promising approach to promoting adolescent health via an intervention that is attractive to schools.

Registration

ISRCTN10751359

Abtract - 187 words

Keywords: adolescent; schools; environment; trial; behaviour

For peer review only

Broader impacts of an intervention to transform school environments: additional analyses from the INCLUSIVE cluster randomised controlled trial

INTRODUCTION

We have previously reported the results of our main trial analyses from the INCLUSIVE cluster randomized controlled trial (RCT) of a multi-component intervention aiming to transform school environments to render these healthier places, reporting effectiveness across multiple health domains.(1) In this paper, we aim to explore the extent to which the intervention 'disrupted' the school 'system' to achieve more impacts.

There is increasing interest in the implications of 'systems' thinking for evaluating health interventions.(2) Interventions can be viewed as 'interruptions' to complex systems, the consequences of which may go beyond the primary and secondary health outcomes assessed by trials. This perspective is of particular relevance for our intervention since this aimed to promote students' health not by improving individual students' knowledge, skills or attitudes, but rather by modifying the overall school environment so that it is more engaging and thus an easier environment in which to choose healthy rather than risky behaviours. Because of our interventions' focus on settings and use of multiple components, it is particularly likely that its impact might have gone beyond our pre-hypothesised primary and secondary outcomes to impact on the school systems' broader functioning.(3) This paper therefore draws on theory to develop and test hypotheses about what the broader impacts of our intervention might have been.

Our intervention aimed to transform the school environment to make this more salutogenic, informed by previous evidence of effective interventions.(4-6) It aimed to do so by: (a) using 'restorative approaches' to address conflict; (b) rendering schools more participative by involving students and staff in an action group to review local data on student experiences and use this to change school policies relating to behaviour management, and lead the intervention; and (c) providing a social and

For peer review only - http://bmjopen.bmj.com/site/about/guidelines.xhtml

emotional skills curriculum for students age 12-15 years.(1, 7) Restorative approaches aim to enable victims to communicate to perpetrators the harms experienced, and enable perpetrators to recognise and take steps to remedy this, and avoid further harms.(8) Restorative approaches include primary prevention of conflict (via 'circle-time', which brings students together to build and maintain relationships) and/or secondary prevention to resolve incidents (such as 'conferencing' to address serious incidents).

The intervention was supported by a theory of change which was informed by an appropriate settings-based social theory: the theory of human functioning and school organisation.(9) We theorised that schools can reduce bullying and aggression by transforming the school environment to build student commitment to learning and sense of belonging in school. It was theorised that this in turn can be achieved by improving relationships between and among staff and students (via the action group and restorative practice), and by better integrating students' academic education and broader personal development (via the curriculum and restorative practice). It was further theorised that by increasing student commitment to and belonging in school, this would reduce student interest and involvement in anti-social peer groups and behaviours.

Our main trial paper examined intervention effects on the primary and secondary outcomes described in the trial protocol. We reported a range of significant intervention effects in terms of reduced bullying victimisation (co-primary outcome) and use of tobacco, alcohol and other drugs, reduced contact with police, as well as improved mental wellbeing, psychological functioning and health-related quality of life among adolescent students (secondary outcomes) at 36 months.(1) We found no significant effect for perpetration of aggression in school (co-primary outcome) or for age of sexual debut, use of contraception at first sex, bullying perpetration or use of NHS services (secondary outcomes). The intervention was implemented with variable fidelity, with this being lower in year 3. Training, action groups and restorative practices but not the curriculum were delivered with good fidelity.(1)

For peer review only - http://bmjopen.bmj.com/site/about/guidelines.xhtml

The first area where we anticipate beneficial broader impacts is cyber-bullying. Our main trial analyses reported effects of the intervention on our primary outcome of reduced bullying victimisation (Gatehouse Bullying Scale, GBS(10)). This is an important result given the prevalence of bullying(11) and its association with concurrent and future physical and mental health harms.(12-18) However, this analysis was insensitive to any effects of the intervention on cyber-bullying. Cyber-bullying is an increasingly prevalent aspect of bullying, associated with significant harms.(19) We did not include this in our list of primary or secondary outcomes because this mostly occurs outside school. However, assessing this would be appropriate given that our intervention is theorised to work by decreasing student interest in anti-social behaviour in general not limited to the school site.

Our second area of exploration is perpetration of aggression. Our main trial analyses found no evidence of effects on our other primary outcome of self-reported perpetration of school-based aggression (Edinburgh Study of Youth Transitions, ESYTC, measure(20)). This was an unexpected finding given the reduction in bullying victimisation, and given the intervention was theorised to reduce bullying victimisation and perpetration of aggression via a common mechanism involving increased student commitment to school and reducing student involvement in anti-social peer groups and behaviours. A systematic review has previously concluded that trials of whole-school interventions addressing violence sometimes find effects on victimisation but not perpetration, possibly because participants under-report perpetration of socially unacceptable behaviours particularly in school.(5) Our student questionnaire also included a measure for students to report their observations of other students perpetrating aggressive behaviours, thus perhaps providing a broader assessment of aggressive behaviours in school and less prone to under-reporting. Therefore, we hypothesise that we will find effects of the intervention on this measure of student-reported observations of other students perpetrating aggressive behaviours at school. Furthermore, our primary measure of perpetration of aggression focused only on school-based behaviours. Because our intervention aimed to reduce students' general involvement in anti-social peer groups and behaviours, rather than merely reducing such behaviours in school, we hypothesise that the intervention would be

For peer review only - http://bmjopen.bmj.com/site/about/guidelines.xhtml

effective in reducing a broader measure of students' own perpetration of aggressive behaviours not specific to school that we included in our questionnaire.

Our third focus for this paper is on impacts of the intervention on the overall functioning of the school system. Interventions effective in reducing bullying and promoting student health are more likely to be scaled up if schools and policy-makers can see evidence that such interventions also reduce school workloads and enhance education.(21) Our theory of change centred on enhancing student commitment to school and reducing student involvement in anti-social behaviours, and we found effects not only for reduced bullying victimisation but also increased student commitment to school.(22) We therefore hypothesise that this will translate into students reporting: feeling safer at school; less participation in school disciplinary procedures; and less truancy.

Our final focus in this paper is on e-cigarette use. As indicated above, we found effects for smoking but our measure focused on the smoking of tobacco rather than use of e-cigarettes. However, we would also expect the intervention to reduce the latter. There are increasing concerns about the increasing prevalence of e-cigarette use among young people with some evidence that this is associated with subsequent increases in smoking tobacco.(23) We therefore hypothesise that rates of use of e-cigarettes are lower among schools in the intervention group.

In summary, we hypothesise that the intervention was effective not only with regard to the primary and secondary outcome measured described in our protocol, but also in promoting a broader range of unintended but beneficial impacts via its disruption of the school system, reducing student-reported: cyber-bullying victimisation and perpetration; observations of other students perpetrating aggressive behaviours at school; own perpetration of aggressive behaviours in and outside school; perceived lack of safety at school; participation in school disciplinary procedures; truancy; and e-cigarette use. Given, our previous finding that intervention effects on primary and secondary outcomes were apparent at 36- but not 24-month follow-up, which is in line with previous evidence that the effects of

For peer review only

For peer review only - http://bmjopen.bmj.com/site/about/guidelines.xhtml

whole-school interventions build over time as they take time to transform the school environment,(6) we hypothesise that this would also apply to the outcomes examined in this paper.

METHODS

Full details of the intervention and trial are reported in full in our protocol and main trial report.(1, 7) We conducted a two-arm parallel repeat cross-sectional cluster RCT of the intervention in 40 secondary schools in south-east England. To be included, schools had government inspections rating of 'requires improvement' or above, and were recruited by the trial team via emails. Our student population consisted of all students: at baseline in 2014 who were at the end of year 7 (11-12 years); who were then in year 9 at interim 24-month follow-up in 2016; and who were in year 10 final 36-month follow-up in 2017. Some students moved schools, hence the study being repeat cross-sectional since all were included in analyses. Students were surveyed using paper questionnaires in classes under exam conditions by trained fieldworkers blinded to allocation. After baseline surveys, schools were allocated 1:1 to intervention or control by computer-generated random numbers stratified by: single-sex versus mixed-sex school; school-level student free-school-meal eligibility (0-23%; >23%) indicating poverty; and General Certificate of Secondary Education results accounting for student baseline attainment (above/below the median score for England of 1000).

The intervention involved all staff in intervention schools receiving training to use restorative practice to prevent and address student conflicts. Approximately 5-10 key staff per school were trained in-depth to deliver restorative conferences dealing with more serious incidents. All schools received a manual to guide the convening and running of a school action group comprising at least six staff and six students, led by a member of the school's senior leadership team. An external facilitator supported action groups in the first two but not the third year of intervention, when they moved to being self-directed. Action groups reviewed anonymised findings from the school's baseline survey to understand local needs, and aimed to coordinate the intervention and revise policies so that these supported use of restorative practice. Schools were provided with materials to guide delivery of a

For peer review only - http://bmjopen.bmj.com/site/about/guidelines.xhtml

social and emotional skills curriculum for students in years 8-10 to receive 5-10 hours teaching per year. The curriculum addressed bullying and aggression but not specific to a particular setting such as school or online. Schools in the control group continued with usual practice.

Our measures analysed in this paper are described in table 1. Each of these were included in student questionnaires used to assess trial outcomes but did not form part of our specified trial outcomes. We adapted Smith and colleagues' measure of cyber-bullying perpetration and victimisation.(24) We developed a new single-item measure of student-reported observations of other students perpetrating aggressive behaviours at school, where students indicated which behaviours they had observed at school to provide a quantitative measure scored 0-7. We examined students' own perpetration of aggressive behaviours in or outside school using a modified four-item version of the ESYTC measure of anti-social behaviours.(20) Students reported which behaviours they had engaged in to give a quantitative score 0-4. We assessed perceived lack of school safety using a single item derived from the Healthy School Ethos (HSE) study.(25) We assessed student participation in school disciplinary procedures using the six-item ESYTC measure of school discipline.(20) This assessed students' frequency of engagement (never; one or two times; three or four times; five or more times) with six disciplinary procedures to provide a quantitative score 0-18. We assessed school truancy using a student-reported single item measure previously used in the Ripple trial.(26) We developed a new single-item measure of e-cigarette use.

PATIENT AND PUBLIC INVOLVEMENT

The trial involved young people from The National Children's Bureau Young Researchers' Group (YRG) in advising on intervention and research methods during three meetings during the set-up phase. School action groups comprised part of the intervention and enabled students to participate in planning and coordinating intervention activities.

For peer review only - http://bmjopen.bmj.com/site/about/guidelines.xhtml

TABLE 1: OUTCOME MEASURES

Outcome measure	Question	Responses	Source	Variable
Cyber-bullying victimisation	Have you been bullied through mobile phone use or on the internet in the last three months?	No I haven't<br>Yes, once or twice<br>Yes, two or three times a month<br>Yes, about once a week<br>Yes, several times a week or more	Adapted from DAPHNE measure of cyber-bullying	Binary any yes /no
Cyber-bullying perpetration	Have you ever bullied anyone else using your mobile phone or using the internet?	No I haven't<br>Yes, once or twice<br>Yes, two or three times a month<br>Yes, about once a week Yes, several times a week or more	Adapted from DAPHNE measure of cyber-bullying	Binary any yes/no
Student-reported observations of other students perpetrating aggressive behaviours at school	Which of the following have you seen happen at this school in the last 3 months of school	Boys fighting<br>Girls fighting<br>Someone threatening someone<br>A student trying to hurt another student<br>Someone robbing money or a mobile phone<br>Someone letting off a firework<br>Someone carrying a knife	New	Score out of 7 (point per item)
Perpetration of anti-social behaviour in or outside school	During the last 3 months of school:<br>Did you ever carry a knife or other weapon with you for protection or in case it was needed in a fight?<br>Did you use force, threats or a weapon to steal money or something else from somebody?<br>Did you damage or destroy property that did not belong to you on purpose	No<br>Yes	Adapted from ESYTC measure of anti-social behaviour	Score out of 4 (4 items with each n/y 0-1)

For peer review only - http://bmjopen.bmj.com/site/about/guidelines.xhtml

		(e.g. windows, cars or street lights)?			
		Did you ever set fire or try to set fire to something on purpose (e.g. bus shelter, shop, etc.)?			
Participation in school disciplinary procedures	During the last 3 months of school how often did these things happen to you because of something you had done wrong?	The school got in touch with my parents by letter or telephone about an incident	0 times<br>1 or 2 times<br>3 or 4 times<br>5 or more times	ESYTC measure of school discipline	Score out of 18 (6 items with each scored 0-3)
		I got a punishment and my parents were informed about that			
		I was given detention			
		I was sent to the head of year, deputy head or head teacher for my behaviour			
		I was put on a conduct/behaviour sheet			
		I was given extra homework to do			
Truancy	During the last 3 months of school have you skipped / bunked off school?		No<br>Yes	Ripple measure of truancy	Binary y/n
E-cigarette use	Which of the following best describes you?		I currently smoke e cigarettes<br>I have tried e cigarettes in the past 12 months but do not currently smoke them	New	Binary – ever/ never

For peer review only - http://bmjopen.bmj.com/site/about/guidelines.xhtml

		I have tried e cigarettes longer than 12 months ago but do not currently smoke them<br>I have never tried e-cigarettes		
Perceived lack of school safety	Do you feel safe at this school?	All of the time<br>Most of the time Some of the time<br>Never	HSE measure of school safety	Binary – some of the time/never versus other options

For peer review only

For peer review only - http://bmjopen.bmj.com/site/about/guidelines.xhtml

As with our analysis of primary and secondary outcomes, our analyses of outcomes in this paper were intention-to-treat, including all schools and participants at each wave. Each outcome measure was analysed using a separate mixed-model with the measure from each time point treated as a repeated measures. Fixed effects of time (baseline, 24 months and 36 months) and the interaction between arm and time were specified, and estimated baseline measures were constrained to be identical in the two arms of the trial. This is equivalent to adjusting for baseline but enables data from all participants to contribute to the analysis, even where there are missing data at follow up. We specified random effects for school and participants, to allow for correlations within schools and repeated measures within participants. We undertook analyses adjusted for baseline measures of outcomes, sex, ethnicity, socioeconomic status (Family Affluence Scale, FAS) as well as for the school stratifying factors.

We used appropriate multilevel models to examine the effects of the intervention. For quantitative measures, we used mixed linear-regression models with random effects at the level of participants and schools to estimate adjusted mean differences (MD) between arms. For binary outcomes, we used mixed effects logistic regression models, with random effects for schools and indiviuals, reporting unadjusted and adjusted odds ratios (OR). Evidence for any moderation of intervention effects on our outcomes by student sex and socioeconomic status (Family Affluence Scale) was assessed by Wald tests for the treatment by subgroup interaction terms. We also calculated Cronbach's alphas to assess the inter-item reliability of our multi-question measures of student perpetration of aggressive behaviours in or outside school and student participation in school disciplinary procedures.

The trial was prospectively registered as ISRCTN10751359 with the ISRCTN Registry on 30/01/2014, and approved by the UCL Ethics Committee (ref 5248/001). Informed consent was sought from head-teachers for randomisation and intervention, and from students, deemed competent by schools to do so, for participation in surveys. Parents were informed about the study and could withdraw their children from research activities.

For peer review only - http://bmjopen.bmj.com/site/about/guidelines.xhtml

RESULTS

In total, 6,667 students in the 40 schools provided data at baseline, representing a participation rate of 93.6% of registered students (92.9% in intervention arm; 94.3% control arm). Student characteristics and baseline reports of the outcomes examined in this paper are reported in table 2, with good balance between arms.

TABLE 2: CHARACTERISTICS OF SCHOOLS AND STUDENTS AT BASELINE BY TRIAL ARM

**School characteristics**	**Control 20 schools**	**Intervention 20 schools**	**Overall 40 schools**
School sex mix, n (%)			
Mixed	15 (75·0)	15 (75·0)	30 (75·0)
Girls	3 (15·0)	4 (20·0)	7 (17·5)
Boys	2 (10·0)	1 (5·0)	3 (7·5)
Ofsted rating*, n (%)			
Excellent	5 (25·0)	6 (30·0)	11 (27·5)
Good	13 (65·0)	12 (60·0)	25 (62·5)
Requires improvement	2 (10·0)	2 (10·0)	4 (10·0)
Value added score, mean (SD)	1,003 (24·8)	1,004 (20·4)	1,003 (22·4)
Proportion of students on free school means, mean (SD)	36 (18·0)	35 (22·0)	36 (20·0)
Income Deprivation Affecting Children Index (IDACI), mean (SD)	0·26 (0·2)	0·24 (0·2)	0·25 (0·2)
**Student socio-demographic characteristics**	**3,347 students†**	**3,320 students†**	**6,667 students†**
Age, mean (SD)	12 (0·4)	12 (0·4)	12 (0·4)
Sex, n (%)			
Male	1,639 (49·9)	1,464 (44·9)	3,103 (47·3)
Female	1,649 (50·2)	1,804 (55·2)	3,453 (52·7)
Ethnicity, n (%)			
White British	1,391 (41·5)	1,221 (37·3)	2,612 (39·7)
White other	291 (8·8)	273 (8·3)	564 (8·6)
Asian/Asian British	859 (25·9)	786 (24·0)	1,645 (25·0)
Black/Black British	384 (11·6)	535 (16·4)	919 (14·0)
Chinese/Chinese British	11 (0·3)	35 (1·1)	46 (0·7)
Mixed ethnicity	238 (7·2)	224 (6·9)	462 (7·0)
Other	140 (4·2)	198 (6·1)	338 (5·1)
Family affluence scale, mean (SD)	6 (1·8)	6 (1·8)	6 (1·8)
**Student baseline rates of outcomes**			
Cyber-bullying perpetration, n (%)	290 (8·9)	279 (8·6)	569 (8·7)
Cyber-bullying victimisation, n (%)	522 (16·0)	467 (14·5)	989 (15·3)
Truancy, n (%)	189 (5·9)	182 (5·8)	371 (5·8)
E-cigarette use, n (%)	187 (5·8)	131 (4·2)	318 (5·0)
Perceived lack of school safety, n (%)	493 (15·6)	440 (14·5)	933 (15·1)
Student-reported observations of other students perpetrating aggressive behaviours at school, mean (SD)	2·30 (1·61)	2·04 (1·65)	2·17 (1·64)

For peer review only - http://bmjopen.bmj.com/site/about/guidelines.xhtml

For peer review only

School characteristics	Control	Intervention	Overall
Perpetration of aggressive behaviour in/outside school, mean (SD)	0·06 (0·31)	0·06 (0·33)	0·06 (0·32)
Participation in school disciplinary procedures, mean (SD)	2·47 (2·96)	2·39 (3·00)	2·43 (2·98)

* One control school did not have an Ofsted rating
† The number of students who responded at this survey; actual number of responses to each question varies, but item non-response is similar across arms.

All schools participated in the surveys at 24 and 36 months, with student participation rates being similar by arms (figure 1).

Cronbach's alphas for our ESYTC measures of student perpetration of aggressive behaviours in or outside school was 0.55 and for our ESYTC measure of student participation in school disciplinary procedures was 0.79.

Our broader student outcomes at 24 and 36 months are reported in table 3. At 24 months comparing intervention with control schools, we found lower rates of: cyber-bullying victimisation (OR=0.77; 95% confidence interval (CI) 0.61, 0.98; P=0.035) but not perpetration; and e-cigarette use (OR=0.60 95% CI 0.43, 0.83; P=0.002). Students in intervention schools were more likely to report lack of perceived school safety at 24 months than controls (OR=1.38, 95% CI 1.10, 1.75; P=0.006). There was no evidence of difference between arms in: student-reported observations of other students perpetrating aggressive behaviours at school; perpetration of aggressive behaviours in or outside school; or truancy. There was weak to moderate evidence of lower reported participation in school disciplinary procedures in intervention compared with control schools at 24 months (MD=-0.16, 95% CI -0.32, 0.00; P=0.043).

For peer review only - http://bmjopen.bmj.com/site/about/guidelines.xhtml

TABLE 3: OUTCOMES AT 24- AND 36-MONTH FOLLOW-UP

Outcomes	24 months				36 months			
	Control n/N (%) or mean (SD), N	Intervention n/N (%) or mean (SD), N (%)	Unadjusted OR or MD (95% confidence interval), P-value	Adjusted OR or MD (95% confidence interval), P-value	Control n/N (%) or mean (SD)	Intervention n/N (%) or mean (SD), N (%)	Unadjusted OR or MD (95% confidence interval), P-value	Adjusted OR or MD (95% confidence interval), P-value
Cyber-bullying victimisation	443/3116 (14.2%)	340/2993 (11.4%)	OR=0.79 (0.63 to 0.99), 0.041	OR= 0.77 (0.61 to 0.98), 0.035	347/2987 (11.6%)	266/2754 (9.7%)	OR=0.83 (0.65 to 1.05), 0.121	OR=0.80 (0.62 to 1.05), 0.110
Cyber-bullying perpetration	257/3112 (8.3%)	229/2984 (7.7%)	OR=0.93 (0.71 to 1.21), 0.572	OR=0.90 (0.67 to 1.19), 0.450	287/3008 (9.5%)	193/2766 (7.0%)	OR=0.66 (0.51 to 0.87), 0.003	OR=0.65 (0.48 to 0.88), 0.005
Perceived lack of school safety	543/3106 (17.5%)	568/2944 (7.7%)	OR=1.39 (1.11 to 1.74), 0.004	OR=1.39 (1.10 to 1.75), 0.006	601/2919 (20.6%)	532/2682 (19.8%)	OR=1.08 (0.86 to 1.36), 0.495	OR=1.05 (0.83 to 1.34), 0.440
Student-reported observations of other students perpetrating aggressive behaviours at school	2.43 (1.65), 3179	2.12 (1.71), 3063	MD=-0.05 (-0.14 to 0.05), 0.344	MD=-0.08 (-0.18 to 0.01), 0.096	2.23 (1.77), 3069	2.07 (1.76), 2834	MD=0.11 (0.01 to 0.20), 0.030	MD=0.10 (0.00 to 0.20), 0.049
Perpetration of aggressive behaviours in/ outside school	0.10 (0.44), 3149	0.08 (0.39), 3017	MD=-0.014 (-0.037 to 0.009), 0.229	MD=-0.009 (-0.034 to 0.015), 0.456	0.12 (0.50), 3023	0.09 (0.43), 2778	MD=-0.030 (-0.054 to -0.007), 0.012	MD=-0.031 (-0.056 to -0.006), 0.016
Participation in school disciplinary procedures	2.74 (3.28), 3128	2.36 (3.12), 3009	MD=-0.222 (-0.375 to -0.069), 0.004	MD=-0.160 (-0.320 to 0), 0.043	2.55 (3.21), 3017	2.15 (2.94), 2757	MD=-0.326 (-0.484 to -0.169), <0.001	MD=-0.320 (-0.480 to -0.150), <0.001
Truancy	340/3088 (11.0%)	299/2961 (10.1%)	OR=0.93 (0.72 to 1.20), 0.582	OR=0.92 (0.70 to 1.21), 0.551	426/2981 (14.3%)	294/2750 (10.7%)	OR=0.66 (0.51 to 0.85), 0.001	OR=0.64 (0.49 to 0.84), 0.001

For peer review only

For peer review only - http://bmjopen.bmj.com/site/about/guidelines.xhtml

E-cigarette use	558/3061 (18.2%)	352/2913 (12.1%)	OR=0.62 (0.45 to 0.86), 0.004	OR=0.60 (0.43 to 0.83), 0.002	630/2946 (21.4%)	394/2692 (14.6%)	OR=0.60 (0.44 to 0.83), 0.002	OR=0.59 (0.42 to 0.82), 0.002

For peer review only

For peer review only - http://bmjopen.bmj.com/site/about/guidelines.xhtml

At 36 months comparing intervention and control schools, we found reduced rates of: cyber-bullying perpetration (OR=0.65, 95% CI 0.48, 0.88; P=0.005) but not victimisation; perpetration of aggression in or outside school (MD=-0.031 95% CI -0.056, -0.006; P=0.016); participation in school disciplinary procedures (MD=-0.320, 95% CI -0.480, -0.150; P<0.001); truancy (OR=0.64, 95% CI 0.49, 0.84; P=0.001); and e-cigarette use (OR=0.59, 95% CI 0.42, 0.82; P=0.002). There was weak to moderate evidence of lower student-reported observations of other students perpetrating aggressive behaviours at school (MD=0.10, 95% CI 0.00, 0.20; P=0.049). There were no evidence of difference in perceived school safety.

Table 4 presents outcomes and follow-up points for which there was evidence of moderation. We found evidence that intervention effects on cyber-bullying perpetration at 24 months were moderated by student sex, such that effects were larger for boys (P=0.002). Intervention effects on observed aggression by other students at 24 months were moderated by student sex and socioeconomic status, with effects larger for girls (P=0.02) and affluent students (P=0.03). Effects on perceived lack of school safety at 24 months were larger for girls (P=0.001), and at 36 months were larger for students from poorer families (P=0.002). Effects on participations in school disciplinary procedures at 24 and 36 months were larger for boys (P<0.001; P=0.001 respectively). Effects on truancy at 24 months were larger for boys (P=0.015). Effects on e-cigarette use at 24 and 36 months were larger for boys (P=0.014; P<0.001 respectively).

TABLE 4: MODERATION

**Outcome**	**Follow-up (months) where evidence of moderation**	**Moderator**		**Association (95% CI)**	**Interaction P value**
		Variable	***Categories***		
Perpetration cyber-bullying	24	Sex	Boys	OR=0.61 (0.41, 0.89)	0.002
			Girls	1.19 (0.85, 1.67)	
Observed aggression	24	Sex	Boys	MD=0.01 (-0.11, 0.13)	0.02
			Girls	MD=-0.15 (-0.26, -0.04)	

For peer review only - http://bmjopen.bmj.com/site/about/guidelines.xhtml

		Family affluence	Low	MD=-0.18 (-0.62, 0.25)	0.03
			Middle	MD=0.04 (-0.09, 0.18)	
			High	MD=-0.14 (-0.25, -0.03)	
Lack of safety	24	Sex	Boys	OR=0.99 (0.73, 1.35)	0.001
			Girls	OR=1.74 (1.33, 2.27)	
	36	Family affluence	Low	OR=3.07 (0.99-9.54)	0.002
			Middle	OR=0.72 (0.51, 1.01)	
			High	OR=1.23 (0.94, 1.60)	
Participation in school disciplinary procedures	24	Sex	Boys	MD=-0.39 (-0.60, -0.19)	<0.001
			Girls	MD=0.02 (-0.17, 0.20)	
	36	Sex	Boys	MD=-0.55 (-0.76, -0.34)	0.001
			Girls	MD=-0.14 (-0.33, 0.05)	
Truancy	24	Sex	Boys	OR=0.69 (0.48, 0.99)	0.015
			Girls	OR=1.13 (0.83-1.55)	
E-cigarette use	24	Sex	Boys	OR=0.45 (0.30, 0.67)	0.014
			Girls	OR=0.80 (0.54, 1.20	
	36		Boys	OR=0.35 (0.23, 0.53	<0.001
			Girls	OR=0.94 (0.63, 1.40).	

For peer review only

DISCUSSION

Summary of key findings

We report an analysis of broader system impacts on student health and school functioning outcomes of this settings-based intervention aim to render schools more health-promoting environments, which

was previously reported to be effective in reducing bullying victimisation and use of alcohol, tobacco and drugs, as well as promoting mental and physical health.(1) We found evidence at 36 but not 24 months of intervention effects on: cyber-bullying perpetration; student observations of aggression by other students; students' own perpetration of aggressive behaviours in or outside school; truancy; and participation in school disciplinary procedures. There was evidence of an effect on increased student perceptions of lack of school safety at 24 but not 36 months. There was also evidence of an effect at 24 but not 36 months on cyber-bullying victimisation. We found evidence of an effect on e-cigarette use at both time-points.

These findings suggest that our intervention, which aimed to reduce bullying via making schools more engaging environments, and which did not explicitly focus on cyber-bullying, might nonetheless have been effective in reducing this. Our findings also suggest that the intervention might, contrary to the main analyses, have reduced rates of aggression including aggression beyond the school environment. However, results across time-points and measures are somewhat inconsistent, probably as a result of chance. Our intervention also appears to have reduced student use of e-cigarettes, which is an important finding given increasing concerns about this as a gateway to tobacco use.(23) This evidence of additional health impacts provides further evidence in support of the intervention theory of change, that it is possible to improve young people's health across a range of areas by addressing the school as a potentially salutogenic environment rather than merely as a setting for individual-focused health education in classrooms. Furthermore, our finding of broader impacts on school functioning in terms of reduced truancy and student involvement in discipline systems, provides evidence of the knock-on consequences of a health intervention disrupting the school system to achieve impacts in the domain of education.

Intervention effects were moderated in some cases by student sex and family affluence. Effects were larger for boys regarding reduced cyber-bullying perpetration and truancy at 24 months, and reduced participations in school disciplinary procedures and e-cigarette use at 24 and 36. For girls,

For peer review only - http://bmjopen.bmj.com/site/about/guidelines.xhtml

For peer review only

intervention effects were greater regarding reduced observed aggression by other students and decreased perceived school safety at 24 months. Effects for decreased perceived school safety at 36 months were also larger for students from poorer families. Effects on reduced observed aggression at 24 months were larger for affluent students. These findings contrast with moderator analyses for our primary and secondary outcomes, where benefits were generally larger for boys and no different for those from poorer families.(1)

Our finding of increased student perceptions of lack of safety in intervention versus control schools at 24 months, and among poorer students at 36 months, is of concern. We note that this association runs counter to our previous findings for actual rates of bullying victimisation(1) and to the findings in this paper on aggression. This finding may be due to chance. However, it may might be explained by the intervention's focus on bullying and aggression sensitizing students to issues of safety, leading them to feel unsafe at the initial (24 month) follow-up but with this dissipating as the intervention became normalised and exerted positive effects on bullying and aggression.

These moderator analyses add to the evidence from our main analyses that the intervention might generally have been more effective for boys than girls. As with our main trial analyses, there was less evidence for moderation by socioeconomic status. These findings of gender inequity of effects is in line with some previous research suggesting that whole-school interventions, including those to reduce violence, can sometimes be more effective for boys than girls.(1, 4) This might be because, in such interventions, violence and other problem behaviours among boys receive more attention than those experienced by girls.

Strengths and limitations

The study was a rigorously conducted experimental evaluation. These are additional analyses not included in our original protocol. Therefore, caution is required in the interpretation of significant

For peer review only - http://bmjopen.bmj.com/site/about/guidelines.xhtml

findings. However, the analyses are guided by explicit, theory-driven hypotheses, as set out in our introduction, rather than being the product of subjecting all measures to analysis and merely reporting significant findings. We relied on student self-reports as these were less likely than school routine data to be biased by delivery of the intervention. Where possible, we used reliable existing measures. While our measure of student participation in disciplinary procedures had high inter-item reliability, this was lower for our measure of student perpetration of aggressive behaviours in or outside school so our conclusions regarding effects on this outcome should be cautious.

For peer review only

Implications for policy and research

Our findings suggest that the intervention disrupted school systems to achieve a range of unintended but beneficial impacts on student health and school functioning not captured in the main trial analyses.(2, 3) These results suggest that it is possible to achieve public health improvements across a range of outcomes using a single coordinated intervention which focuses on environmental transformation rather than individual behaviour-change. This is important given the impracticality of implementing different interventions for multiple outcomes in schools.(21) Our findings also suggest important benefits for education and school functioning. It appears that the intervention's previously reported effects on reducing bullying victimisation and improving student commitment to school(1, 22) translated into reduced student truancy and participation in school disciplinary procedures. This is especially important evidence for school leaders suggesting the potential educational benefits of whole-school health interventions. Our analyses suggest this intervention worked more effectively for boys than girls. Further research is needed into how to ensure school-based interventions are more equitable, perhaps by ensuring these address less overt forms of student disengagement and conflict.

Figure 1: Trial participants

ACKNOWLEDGEMENTS

For peer review only - http://bmjopen.bmj.com/site/about/guidelines.xhtml

We are grateful to the staff and students of participating schools for their dedication to the intervention and completion of the outcome surveys and process evaluation surveys and interviews. We are very grateful for the advice and support of our Trial Steering Committee and Data Monitoring Committee. All those who contributed significantly to this work are listed in these acknowledgements.

COMPETING INTERESTS

None to declare.

This article has not been submitted and will not be submitted for publication elsewhere while under consideration by BMJ Open. The paper includes data (on response rates and baseline comparisons between intervention and control groups) that was included in the paper cited below. Other analyses are new to this paper.

Bonell C, Allen E, Warren E, McGowan J, Bevilacqua L, Jamal F, et al. Initiating change in the school environment to reduce bullying and aggression: a cluster randomised controlled trial of the Learning Together (LT) intervention in English secondary schools. The Lancet. 2018;392(10163):2452-64.

FUNDING

This work was supported by the National Institute for Health Research (NIHR) in England under its Public Health Research Board (12/153/60) and the Education Endowment Foundation (no grant number). The views expressed in this publication are those of the authors and do not necessarily reflect those of the National Health Service (NHS), the NIHR or the Department of Health for England. The study funders (National Institute for Health Research and Education Endowment

For peer review only - http://bmjopen.bmj.com/site/about/guidelines.xhtml
For peer review only

Foundation played no role in the: study; collection, analysis and interpretation of data; the writing of the report; or the decision to submit the manuscript for publication.

DATA STATEMENT

Anonymised data from the study are available on request.

For peer review only

REFERENCES

1. Bonell C, Allen E, Warren E, McGowan J, Bevilacqua L, Jamal F, et al. Initiating change in the school environment to reduce bullying and aggression: a cluster randomised controlled trial of the Learning Together (LT) intervention in English secondary schools. The Lancet. 2018;392(10163):2452-64.
2. Moore GF, Evans RE, Hawkins J, Littlecott H, Melendez-Torres GJ, Bonell GJ, et al. From complex social interventions to interventions in complex social systems: future directions and unresolved questions for intervention development and evaluation. Evaluation and Program Planning. 2019;;25(1):23-45.
3. Rosas SR. Systems thinking and complexity: considerations for health promoting schools Health Promotion International. 2017;32(2):301-11.
4. Flay BR, Graumlich S, Segawa E, Burns JL, Holliday MY. Effects of 2 prevention programs on high-risk behaviors among African American youth: a randomized trial. Archives of Pediatrics & Adolescent Medicine. 2004;158(4):377-84.
5. Langford R, Bonell CP, Jones HE, Pouliou T, Murphy SM, Waters E, et al. The WHO Health Promoting School framework for improving the health and well-being of students and staff. Cochrane Database of Systematic Reviews 2011, Issue 1 Art No: CD008958. 2014.
6. Patton G, Bond L, Carlin JB, Thomas L, Butler H, Glover S, et al. Promoting social inclusion in schools: group-randomized trial of effects on student health risk behaviour and well-being. American Journal of Public Health. 2006;96(9):1582-7.
7. Bonell C, Allen E, Christie D, Elbourne D, Fletcher A, Grieve R, et al. Initiating change locally in bullying and aggression through the school environment (INCLUSIVE): study protocol for a cluster randomised controlled trial. Trials. 2014;15:381.
8. Morrison B. Restorative justice in schools. In: Elliot E, Gordon RM, editors. New directions in restorative justice: Issues, practices, evaluation. Cullompton, UK: Willan Publishing; 2005. p. 26-52.
9. Markham WA, Aveyard P. A new theory of health promoting schools based on human functioning, school organisation and pedagogic practice. Social Science & Medicine. 2003;56(6):1209-20.
10. Bond L, Wolfe S, Tollit M, Butler H, Patton G. A comparison of the Gatehouse Bullying Scale and the peer relations questionnaire for students in secondary school. Journal of School Health. 2007;77(2):75-9.
11. Inchley J, Currie D, Young T, Samdal O, Torsheim T, Augustson L, et al. Growing up unequal: gender and socioeconomic differences in young people's health and well-being. Copenhagen: WHO Europe, 2016 Contract No.: ISBN 978 92 890 5136 1.
12. Arseneault L. Annual Research Review: The persistent and pervasive impact of being bullied in childhood and adolescence: implications for policy and practice. J Child Psychol Psychiatry. 2017.
13. Hair EC, Park MJ, Ling TJ, Moore KA. Risky behaviors in late adolescence: co-occurrence, predictors, and consequences. J Adolesc Health. 2009;45(3):253-61.
14. Copeland WE, Wolke D, Angold A, Costello EJ. Adult psychiatric outcomes of bullying and being bullied by peers in childhood and adolescence. JAMA Psychiatry. 2013;70(4):419-26.
15. Lereya ST, Copeland WE, Costello EJ, Wolke D. Adult mental health consequences of peer bullying and maltreatment in childhood: two cohorts in two countries. Lancet Psychiatry. 2015;2(6):524-31.
16. Fisher HL, Moffitt TE, Houts RM, Belsky DW, Arseneault L, Caspi A. Bullying victimisation and risk of self harm in early adolescence: longitudinal cohort study. BMJ. 2012;344:e2683.
17. Winsper C, Lereya T, Zanarini M, Wolke D. Involvement in bullying and suicide-related behavior at 11 years: a prospective birth cohort study. J Am Acad Child Adolesc Psychiatry. 2012;51(3):271-82 e3.
18. Fantaguzzi C, Allen E, Miners A, Christie D, Opondo C, Sadique Z, et al. Health-related quality of life associated with bullying and aggression: a cross-sectional study in English secondary schools. Eur J Health Econ. 2017.
19. Bevilacqua L, Shackleton N, Hale D, Allen E, Bond L, Christie D, et al. The role of family and school-level factors in bullying and cyberbullying: a cross-sectional study. BMC Pediatr. 2017;17(1):160.
20. Smith DJ. School experience and delinquency at ages 13 to 16. Edinburgh: Centre for Law and Society, University of Edinburgh; 2006.
21. Bonell C, Humphrey N, Fletcher A, Moore L, Anderson R, Campbell R. Why schools should promote students' health and wellbeing Education policy shouldn't focus solely on academic attainment. British Medical Journal. 2014;348:g3078.
22. Bonell C, Allen E, Opondo C, Warren E, Elbourne D, Sturgess J, et al. Examining intervention mechanisms of action using mediation analysis within a randomised trial of a whole-school health intervention. Journal of Epidemiology and Community Health http://dxdoiorg/101136/jech-2018-211443. 2019.

For peer review only - http://bmjopen.bmj.com/site/about/guidelines.xhtml

23. Barrington-Trimis JL, Kong G, Leventhal AM, Liu F, Mayer M, Boley Cruz T, et al. E-cigarette use and subsequent smoking frequency among adolescents. Pediatrics. 2018;142(6):e20180486.
24. Smith PK, Mahdavi J, Carvalho M, Fisher S, Russell S, Tippett N. Cyberbullying: Its nature and impact in secondary school pupils. Journal of Child Psychology and Psychiatry. 2008;49(376):e385.
25. Bonell C, Sorhaindo A, Allen E, Strange V, Wiggins M, Fletcher A, et al. Pilot multi-method trial of a school-ethos intervention to reduce substance use: building hypotheses about upstream pathways to prevention. Journal of Adolescent Health. 2010;47(6):555-63.
26. Stephenson JM, Strange V, Forrest S, Oakley A, Copas A, Allen E, et al. Pupil-led sex education in England (RIPPLE study): cluster-randomised intervention trial. Lancet. 2004;364(9431):338-46.

For peer review only

For peer review only - http://bmjopen.bmj.com/site/about/guidelines.xhtml

FIGURE 1: TRIAL PARTICIPANTS

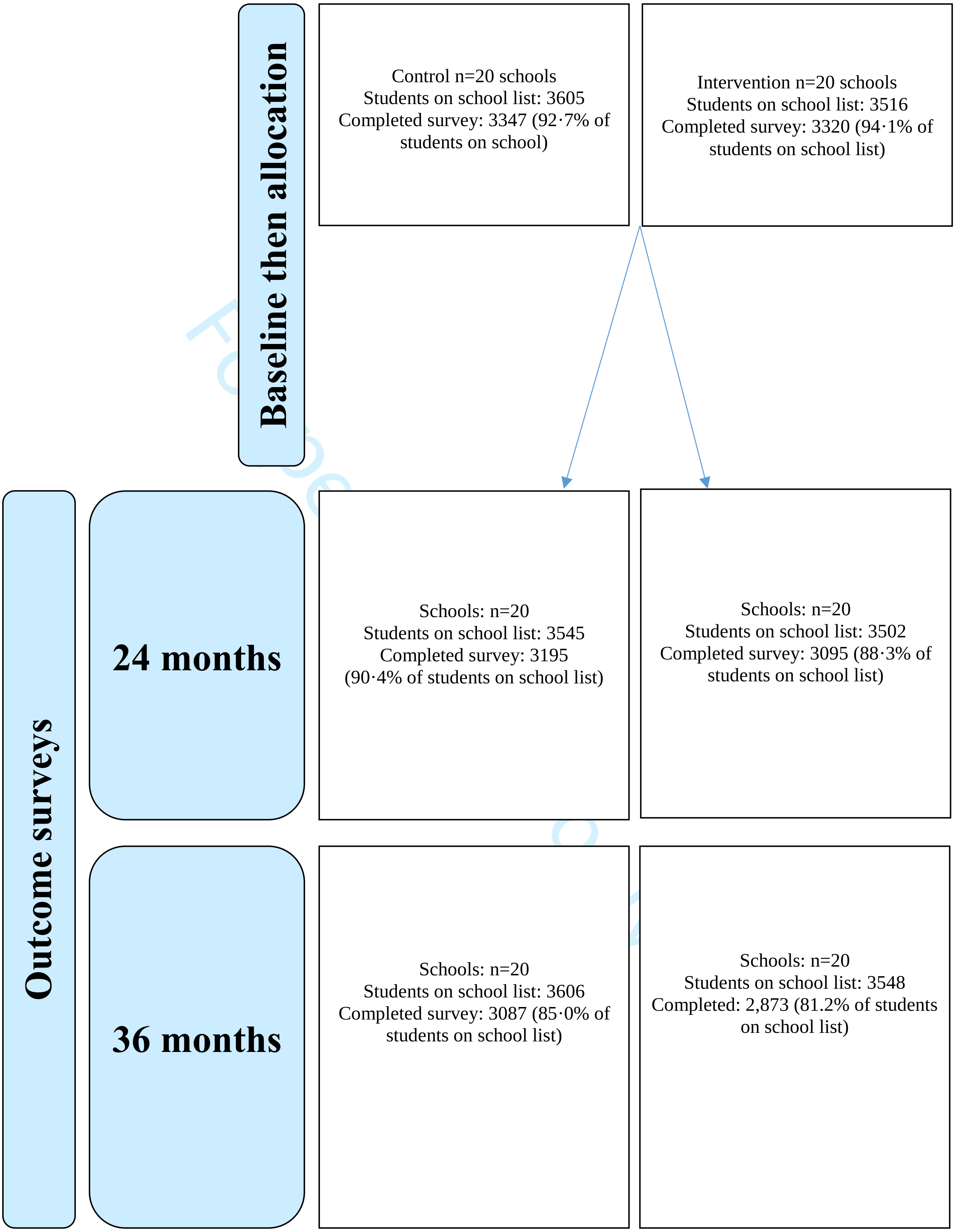

CONSORT 2010 checklist of information to include when reporting a randomised trial*

Section/Topic	Item No	Checklist item	Reported on page No
**Title and abstract**			
	1a	Identification as a randomised trial in the title	1,3,5
	1b	Structured summary of trial design, methods, results, and conclusions (for specific guidance see CONSORT for abstracts)	3,4
**Introduction**			
Background and objectives	2a	Scientific background and explanation of rationale	5-8
	2b	Specific objectives or hypotheses	8
**Methods**			
Trial design	3a	Description of trial design (such as parallel, factorial) including allocation ratio	8
	3b	Important changes to methods after trial commencement (such as eligibility criteria), with reasons	NA
Participants	4a	Eligibility criteria for participants	8,9
	4b	Settings and locations where the data were collected	9
Interventions	5	The interventions for each group with sufficient details to allow replication, including how and when they were actually administered	9
Outcomes	6a	Completely defined pre-specified primary and secondary outcome measures, including how and when they were assessed	Additional analysis. 9-13
	6b	Any changes to trial outcomes after the trial commenced, with reasons	NA
Sample size	7a	How sample size was determined	reference to main trial report
	7b	When applicable, explanation of any interim analyses and stopping guidelines	NA
Randomisation:			9 + reference to main trial report
Sequence generation	8a	Method used to generate the random allocation sequence	
	8b	Type of randomisation; details of any restriction (such as blocking and block size)	9
Allocation concealment	9	Mechanism used to implement the random allocation sequence (such as sequentially numbered containers), describing any steps taken to conceal the sequence until interventions were assigned	9 + reference to main trial

For peer review only

mechanism			report
Implementation	10	Who generated the random allocation sequence, who enrolled participants, and who assigned participants to interventions	9 + reference to main trial report
Blinding	11a	If done, who was blinded after assignment to interventions (for example, participants, care providers, those assessing outcomes) and how	9
	11b	If relevant, description of the similarity of interventions	NA
Statistical methods	12a	Statistical methods used to compare groups for primary and secondary outcomes	14 + reference to main trial report
	12b	Methods for additional analyses, such as subgroup analyses and adjusted analyses	14
**Results**			
Participant flow (a diagram is strongly recommended)	13a	For each group, the numbers of participants who were randomly assigned, received intended treatment, and were analysed for the primary outcome	15, figure 1 + reference to main trial report
	13b	For each group, losses and exclusions after randomisation, together with reasons	15, 16 + reference to main trial report
Recruitment	14a	Dates defining the periods of recruitment and follow-up	9
	14b	Why the trial ended or was stopped	NA
Baseline data	15	A table showing baseline demographic and clinical characteristics for each group	Table 2
Numbers analysed	16	For each group, number of participants (denominator) included in each analysis and whether the analysis was by original assigned groups	Tables 3-4
Outcomes and estimation	17a	For each primary and secondary outcome, results for each group, and the estimated effect size and its precision (such as 95% confidence interval)	Table 3
	17b	For binary outcomes, presentation of both absolute and relative effect sizes is recommended	reference to main trial report
Ancillary analyses	18	Results of any other analyses performed, including subgroup analyses and adjusted analyses, distinguishing pre-specified from exploratory	Table 3

For peer review only - http://bmjopen.bmj.com/site/about/guidelines.xhtml
For peer review only

Harms	19	All important harms or unintended effects in each group (for specific guidance see CONSORT for harms)	reference to main trial report
**Discussion**			
Limitations	20	Trial limitations, addressing sources of potential bias, imprecision, and, if relevant, multiplicity of analyses	23
Generalisability	21	Generalisability (external validity, applicability) of the trial findings	23
Interpretation	22	Interpretation consistent with results, balancing benefits and harms, and considering other relevant evidence	23
**Other information**			
Registration	23	Registration number and name of trial registry	14
Protocol	24	Where the full trial protocol can be accessed, if available	14
Funding	25	Sources of funding and other support (such as supply of drugs), role of funders	24-25

*We strongly recommend reading this statement in conjunction with the CONSORT 2010 Explanation and Elaboration for important clarifications on all the items. If relevant, we also recommend reading CONSORT extensions for cluster randomised trials, non-inferiority and equivalence trials, non-pharmacological treatments, herbal interventions, and pragmatic trials. Additional extensions are forthcoming: for those and for up to date references relevant to this checklist, see www.consort-statement.org.

For peer review only

For peer review only - http://bmjopen.bmj.com/site/about/guidelines.xhtml

BMJ Open

Broader impacts of an intervention to transform school environments on student behaviour and school functioning: post hoc analyses from the INCLUSIVE cluster randomised controlled trial

Journal:	*BMJ Open*
Manuscript ID	bmjopen-2019-031589.R2
Article Type:	Original research
Date Submitted by the Author:	05-Mar-2020
Complete List of Authors:	Bonell, Christopher; London School of Hygiene and Tropical Medicine, Public Health and Policy<br>Dodd, Matthew; London School of Hygiene and Tropical Medicine, Public Health and Policy<br>Allen, Elizabeth; London School of Hygiene & Tropical Medicine, Department of Medical Statistics<br>Bevilacqua, Leonardo; University College London<br>McGowan, Jennifer; University College London<br>Opondo, Charles; London School of Hygiene and Tropical Medicine<br>Sturgess, Joanna; London School of Hygiene and Tropical Medicine, Department of Population Health<br>Elbourne, Diana; LSHTM, EPH<br>Warren, Emily; London School of Hygiene & Tropical Medicine, Faculty of Epidemiology and Population Health<br>Viner, Russell; UCL Institute of child health, Population, policy and practice research programme
**Primary Subject Heading**:	Public health
Secondary Subject Heading:	Epidemiology, Smoking and tobacco
Keywords:	PUBLIC HEALTH, EPIDEMIOLOGY, STATISTICS & RESEARCH METHODS

SCHOLARONE™
Manuscripts

I, the Submitting Author has the right to grant and does grant on behalf of all authors of the Work (as defined in the below author licence), an exclusive licence and/or a non-exclusive licence for contributions from authors who are: i) UK Crown employees; ii) where BMJ has agreed a CC-BY licence shall apply, and/or iii) in accordance with the terms applicable for US Federal Government officers or employees acting as part of their official duties; on a worldwide, perpetual, irrevocable, royalty-free basis to BMJ Publishing Group Ltd ("BMJ") its licensees and where the relevant Journal is co-owned by BMJ to the co-owners of the Journal, to publish the Work in this journal and any other BMJ products and to exploit all rights, as set out in our licence.

The Submitting Author accepts and understands that any supply made under these terms is made by BMJ to the Submitting Author unless you are acting as an employee on behalf of your employer or a postgraduate student of an affiliated institution which is paying any applicable article publishing charge ("APC") for Open Access articles. Where the Submitting Author wishes to make the Work available on an Open Access basis (and intends to pay the relevant APC), the terms of reuse of such Open Access shall be governed by a Creative Commons licence – details of these licences and which Creative Commons licence will apply to this Work are set out in our licence referred to above.

Other than as permitted in any relevant BMJ Author's Self Archiving Policies, I confirm this Work has not been accepted for publication elsewhere, is not being considered for publication elsewhere and does not duplicate material already published. I confirm all authors consent to publication of this Work and authorise the granting of this licence.

For peer review only

Broader impacts of an intervention to transform school environments on student behaviour and school functioning: post hoc analyses from the INCLUSIVE cluster randomised controlled trial

1. Chris Bonell PhD[a*]
2. Matthew Dodd MSc[b]
3. Elizabeth Allen PhD[b]
4. Leonardo Bevilacqua, MSc[c]
5. Jennifer McGowan PhD[c]
6. Charles Opondo PhD[b]
7. Joanna Sturgess MSc[b]
8. Diana Elbourne PhD[b]
9. Emily Warren MSc[a]
10. Russell M. Viner PhD[c]

* Corresponding author: chris.bonell@lshtm.ac.uk; +44 (0)20 7612 7918 (no fax)

a) Department of Public Health, Environments and Society, London School of Hygiene and Tropical Medicine, 15-17 Tavistock Place, London WC1H 9SH
b) Department of Medical Statistics, London School of Hygiene and Tropical Medicine, Keppel Street, London WC1E 7HT
c) UCL Great Ormond St. Institute of Child Health, University College London, 30 Guilford St. London QC1N 1EH, UK

Words: 3,495 words

References: 26

Figure: 1

Tables: 5

For peer review only - http://bmjopen.bmj.com/site/about/guidelines.xhtml

Keywords: school; adolescent health; smoking; violence

Author contributions

All those who contributed significantly to this work are listed as authors. CB and RV directed the trial from which the data are drawn. CB, MD, EA and CO designed the analysis for this paper. MD implemented this design and undertook the analysis. DE provided additional statistical expertise. CB wrote the first draft of the paper which was then edited and commented on by MD, EA, LB, JM, CO, JS, DE, EW and RV.

For peer review only

For peer review only - http://bmjopen.bmj.com/site/about/guidelines.xhtml

Broader impacts of an intervention to transform school environments on student behaviour and school functioning: post hoc analyses from the INCLUSIVE cluster randomised controlled trial

ABSTRACT

Background

We have previously reported benefits for reduced bullying, smoking, alcohol and other drug use and mental health from a trial of 'Learning Together', an intervention which aimed to modify school environments and implement restorative practice and a social and emotional skills curriculum.

Objectives

To conduct post hoc theory-driven analyses of broader impacts.

Design

Cluster randomized trial

Settings

40 state secondary schools in southern England

Participants

Students age 11/12 years at baseline.

Outcomes

Student self-reported measures at 24 and 36 months of: cyber-bullying victimisation and perpetration; observations of other students perpetrating aggressive behaviours at school; own perpetration of aggressive behaviours in and outside school; perceived lack of safety at school; participation in school disciplinary procedures; truancy; and e-cigarette use.

Results

For peer review only

For peer review only - http://bmjopen.bmj.com/site/about/guidelines.xhtml

We found evidence of multiple impacts on other health (reduced e-cigarette use, cyber-bullying perpetration, perpetration of aggressive behaviours) and educational (reduced participation in school disciplinary procedures and truancy) outcomes.

Conclusion

These analyses suggest the intervention was effective in bringing about a broader range of beneficial outcomes, adding to the evidence that the intervention is a promising approach to promoting adolescent health via an intervention that is attractive to schools.

Registration

ISRCTN10751359

Abtract - 187 words

Keywords: adolescent; schools; environment; trial; behaviour

Strengths and limitations

- The study was a rigorously conducted experimental evaluation.
- These are additional analyses not included in our original protocol so caution is required in the interpretation of significant findings.
- However, the analyses are guided by explicit, theory-driven hypotheses, as set out in our introduction, rather than being the product of subjecting all measures to analysis and merely reporting significant findings.
- We relied on student self-reports as these were less likely than school routine data to be biased by delivery of the intervention.
- While our measure of student participation in disciplinary procedures had high inter-item reliability, this was lower for our measure of student perpetration of aggressive behaviours in or outside school so our conclusions regarding effects on this outcome should be cautious.

For peer review only

Broader impacts of an intervention to transform school environments: post hoc analyses from the INCLUSIVE cluster randomised controlled trial

INTRODUCTION

We have previously reported the results of our main trial analyses from the INCLUSIVE cluster randomized controlled trial (RCT) of a multi-component intervention aiming to transform school environments to render these healthier places, reporting effectiveness across multiple health domains.(1) In this paper, we aim to explore the extent to which the intervention 'disrupted' the school 'system' to achieve more impacts.

There is increasing interest in the implications of 'systems' thinking for evaluating health interventions.(2) Interventions can be viewed as 'interruptions' to complex systems, the consequences of which may go beyond the primary and secondary health outcomes assessed by trials. This perspective is of particular relevance for our intervention since this aimed to promote students' health not by improving individual students' knowledge, skills or attitudes, but rather by modifying the overall school environment so that it is more engaging and thus an easier environment in which to choose healthy rather than risky behaviours. Because of our interventions' focus on settings and use of multiple components, it is particularly likely that its impact might have gone beyond our pre-hypothesised primary and secondary outcomes to impact on the school systems' broader functioning.(3) This paper therefore draws on theory to develop and test hypotheses about what the broader impacts of our intervention might have been.

Our intervention aimed to transform the school environment to make this more salutogenic, informed by previous evidence of effective interventions.(4-6) It aimed to do so by: (a) using 'restorative approaches' to address conflict; (b) rendering schools more participative by involving students and staff in an action group to review local data on student experiences and use this to change school policies relating to behaviour management, and lead the intervention; and (c) providing a social and

For peer review only - http://bmjopen.bmj.com/site/about/guidelines.xhtml

emotional skills curriculum for students age 12-15 years.(1, 7) Restorative approaches aim to enable victims to communicate to perpetrators the harms experienced, and enable perpetrators to recognise and take steps to remedy this, and avoid further harms.(8) Restorative approaches include primary prevention of conflict (via 'circle-time', which brings students together to build and maintain relationships) and/or secondary prevention to resolve incidents (such as 'conferencing' to address serious incidents).

The intervention was supported by a theory of change which was informed by an appropriate settings-based social theory: the theory of human functioning and school organisation.(9) We theorised that schools can reduce bullying and aggression by transforming the school environment to build student commitment to learning and sense of belonging in school. It was theorised that this in turn can be achieved by improving relationships between and among staff and students (via the action group and restorative practice), and by better integrating students' academic education and broader personal development (via the curriculum and restorative practice). It was further theorised that by increasing student commitment to and belonging in school, this would reduce student interest and involvement in anti-social peer groups and behaviours.

Our main trial paper examined intervention effects on the primary and secondary outcomes described in the trial protocol. We reported a range of significant intervention effects in terms of reduced bullying victimisation (co-primary outcome) and use of tobacco, alcohol and other drugs, reduced contact with police, as well as improved mental wellbeing, psychological functioning and health-related quality of life among adolescent students (secondary outcomes) at 36 months (table 1).(1) We found no significant effect for perpetration of aggression in school (co-primary outcome) or for age of sexual debut, use of contraception at first sex, bullying perpetration or use of NHS services (secondary outcomes). The intervention was implemented with variable fidelity, with this being lower in year 3. Training, action groups and restorative practices but not the curriculum were delivered with good fidelity.(1)

For peer review only - http://bmjopen.bmj.com/site/about/guidelines.xhtml

TABLE 1: OOUTCOMES ASSESSED IN THE MAIN TRIAL PAPER AND THIS POST HOC ANALYSIS

**Outcomes assessed in main trial paper**	**Evidence of significant beneficial effect on this outcome**	**Outcomes assessed in this post hoc analysis**
Bullying victimisation	√	Cyberbullying victimisation
Aggression perpetration in school		Cyberbullying perpetration
Health-related quality of life	√	Observing other students perpetrating aggression in school
Mental wellbeing	√	Aggression perpetration either in or out of school
Psychological problems	√	Perceived lack of safety at school
Bullying perpetration		Participation in school discipline procedures
Cigarette smoking	√	Truancy
Alcohol use	√	E-cigarette use
Drunkenness	√	
Illicit drug use	√	
Age of sexual debut		
Contraception at first sex		
NHS service use		
Police contact	√	

For peer review only - http://bmjopen.bmj.com/site/about/guidelines.xhtml

The first area where we anticipate beneficial broader impacts is cyber-bullying. Our main trial analyses reported effects of the intervention on our primary outcome of reduced bullying victimisation (Gatehouse Bullying Scale, GBS(10)). This is an important result given the prevalence of bullying(11) and its association with concurrent and future physical and mental health harms.(12-18) However, this analysis was insensitive to any effects of the intervention on cyber-bullying. Cyber-bullying is an increasingly prevalent aspect of bullying, associated with significant harms.(19) We did not include this in our list of primary or secondary outcomes because this mostly occurs outside school. However, assessing this would be appropriate given that our intervention is theorised to work by decreasing student interest in anti-social behaviour in general not limited to the school site.

Our second area of exploration is perpetration of aggression. Our main trial analyses found no evidence of effects on our other primary outcome of self-reported perpetration of school-based aggression (Edinburgh Study of Youth Transitions, ESYTC, measure(20)). This was an unexpected finding given the reduction in bullying victimisation, and given the intervention was theorised to reduce bullying victimisation and perpetration of aggression via a common mechanism involving increased student commitment to school and reducing student involvement in anti-social peer groups and behaviours. A systematic review has previously concluded that trials of whole-school interventions addressing violence sometimes find effects on victimisation but not perpetration, possibly because participants under-report perpetration of socially unacceptable behaviours particularly in school.(5) Our student questionnaire also included a measure for students to report their observations of other students perpetrating aggressive behaviours, thus perhaps providing a broader assessment of aggressive behaviours in school and less prone to under-reporting. Therefore, we hypothesise that we will find effects of the intervention on this measure of student-reported observations of other students perpetrating aggressive behaviours at school. Furthermore, our primary measure of perpetration of aggression focused only on school-based behaviours. Because our intervention aimed to reduce students' general involvement in anti-social peer groups and behaviours, rather than merely reducing such behaviours in school, we hypothesise that the intervention would be

For peer review only - http://bmjopen.bmj.com/site/about/guidelines.xhtml

For peer review only

effective in reducing a broader measure of students' own perpetration of aggressive behaviours not specific to school that we included in our questionnaire.

Our third focus for this paper is on impacts of the intervention on the overall functioning of the school system. Interventions effective in reducing bullying and promoting student health are more likely to be scaled up if schools and policy-makers can see evidence that such interventions also reduce school workloads and enhance education.(21) Our theory of change centred on enhancing student commitment to school and reducing student involvement in anti-social behaviours, and we found effects not only for reduced bullying victimisation but also increased student commitment to school.(22) We therefore hypothesise that this will translate into students reporting: feeling safer at school; less participation in school disciplinary procedures; and less truancy.

Our final focus in this paper is on e-cigarette use. As indicated above, we found effects for smoking but our measure focused on the smoking of tobacco rather than use of e-cigarettes. However, we would also expect the intervention to reduce the latter. There are increasing concerns about the increasing prevalence of e-cigarette use among young people with some evidence that this is associated with subsequent increases in smoking tobacco.(23) We therefore hypothesise that rates of use of e-cigarettes are lower among schools in the intervention group.

In summary, we hypothesise that the intervention was effective not only with regard to the primary and secondary outcome measured described in our protocol, but also in promoting a broader range of unintended but beneficial impacts via its disruption of the school system, reducing student-reported: cyber-bullying victimisation and perpetration; observations of other students perpetrating aggressive behaviours at school; own perpetration of aggressive behaviours in and outside school; perceived lack of safety at school; participation in school disciplinary procedures; truancy; and e-cigarette use (table 1). Given, our previous finding that intervention effects on primary and secondary outcomes were apparent at 36- but not 24-month follow-up, which is in line with previous evidence that the effects of

For peer review only - http://bmjopen.bmj.com/site/about/guidelines.xhtml

whole-school interventions build over time as they take time to transform the school environment,(6) we hypothesise that this would also apply to the outcomes examined in this paper.

METHODS

Full details of the intervention and trial are reported in full in our protocol and main trial report.(1, 7) We conducted a two-arm parallel repeat cross-sectional cluster RCT of the intervention in 40 secondary schools in south-east England. To be included, schools had government inspections rating of 'requires improvement' or above, and were recruited by the trial team via emails. Our student population consisted of all students: at baseline in 2014 who were at the end of year 7 (11-12 years); who were then in year 9 at interim 24-month follow-up in 2016; and who were in year 10 final 36-month follow-up in 2017. Some students moved schools, hence the study being repeat cross-sectional since all were included in analyses. Students were surveyed using paper questionnaires in classes under exam conditions by trained fieldworkers blinded to allocation. After baseline surveys, schools were allocated 1:1 to intervention or control by computer-generated random numbers stratified by: single-sex versus mixed-sex school; school-level student free-school-meal eligibility (0-23%; >23%) indicating poverty; and General Certificate of Secondary Education results accounting for student baseline attainment (above/below the median score for England of 1000).

The intervention involved all staff in intervention schools receiving training to use restorative practice to prevent and address student conflicts. Approximately 5-10 key staff per school were trained in-depth to deliver restorative conferences dealing with more serious incidents. All schools received a manual to guide the convening and running of a school action group comprising at least six staff and six students, led by a member of the school's senior leadership team. An external facilitator supported action groups in the first two but not the third year of intervention, when they moved to being self-directed. Action groups reviewed anonymised findings from the school's baseline survey to understand local needs, and aimed to coordinate the intervention and revise policies so that these supported use of restorative practice. Schools were provided with materials to guide delivery of a

For peer review only - http://bmjopen.bmj.com/site/about/guidelines.xhtml

social and emotional skills curriculum for students in years 8-10 to receive 5-10 hours teaching per year. The curriculum addressed bullying and aggression but not specific to a particular setting such as school or online. Schools in the control group continued with usual practice.

Our measures analysed in this paper are described in table 2. Each of these were included in student questionnaires used to assess trial outcomes but did not form part of our specified trial outcomes. We adapted Smith and colleagues' measure of cyber-bullying perpetration and victimisation.(24) We developed a new single-item measure of student-reported observations of other students perpetrating aggressive behaviours at school, where students indicated which behaviours they had observed at school to provide a quantitative measure scored 0-7. We examined students' own perpetration of aggressive behaviours in or outside school using a modified four-item version of the ESYTC measure of anti-social behaviours.(20) Students reported which behaviours they had engaged in to give a quantitative score 0-4. We assessed perceived lack of school safety using a single item derived from the Healthy School Ethos (HSE) study.(25) We assessed student participation in school disciplinary procedures using the six-item ESYTC measure of school discipline.(20) This assessed students' frequency of engagement (never; one or two times; three or four times; five or more times) with six disciplinary procedures to provide a quantitative score 0-18. We assessed school truancy using a student-reported single item measure previously used in the Ripple trial.(26) We developed a new single-item measure of e-cigarette use.

PATIENT AND PUBLIC INVOLVEMENT

The trial involved young people from The National Children's Bureau Young Researchers' Group (YRG) in advising on intervention and research methods during three meetings during the set-up phase. School action groups comprised part of the intervention and enabled students to participate in planning and coordinating intervention activities.

For peer review only - http://bmjopen.bmj.com/site/about/guidelines.xhtml

TABLE 2: OUTCOME MEASURES

Outcome measure	Question		Responses	Source	Variable
Cyber-bullying victimisation	Have you been bullied through mobile phone use or on the internet in the last three months?		No I haven't<br>Yes, once or twice<br>Yes, two or three times a month<br>Yes, about once a week<br>Yes, several times a week or more	Adapted from DAPHNE measure of cyber-bullying	Binary any yes /no
Cyber-bullying perpetration	Have you ever bullied anyone else using your mobile phone or using the internet?		No I haven't<br>Yes, once or twice<br>Yes, two or three times a month<br>Yes, about once a week Yes, several times a week or more	Adapted from DAPHNE measure of cyber-bullying	Binary any yes/no
Student-reported observations of other students perpetrating aggressive behaviours at school	Which of the following have you seen happen at this school in the last 3 months of school		Boys fighting<br>Girls fighting<br>Someone threatening someone<br>A student trying to hurt another student<br>Someone robbing money or a mobile phone<br>Someone letting off a firework<br>Someone carrying a knife	New	Score out of 7 (point per item)
Perpetration of anti-social behaviour in or outside school	During the last 3 months of school	Did you ever carry a knife or other weapon with you for protection or in case it was needed in a fight?<br>Did you use force, threats or a weapon to steal money or something else from somebody?<br>Did you damage or destroy property that did not belong to you on purpose	No<br>Yes	Adapted from ESYTC measure of anti-social behaviour	Score out of 4 (4 items with each n/y 0-1)

For peer review only

For peer review only - http://bmjopen.bmj.com/site/about/guidelines.xhtml

		(e.g. windows, cars or street lights)?			
		Did you ever set fire or try to set fire to something on purpose (e.g. bus shelter, shop, etc.)?			
Participation in school disciplinary procedures	During the last 3 months of school how often did these things happen to you because of something you had done wrong?	The school got in touch with my parents by letter or telephone about an incident	0 times<br>1 or 2 times<br>3 or 4 times<br>5 or more times	ESYTC measure of school discipline	Score out of 18 (6 items with each scored 0-3)
		I got a punishment and my parents were informed about that			
		I was given detention			
		I was sent to the head of year, deputy head or head teacher for my behaviour			
		I was put on a conduct/behaviour sheet			
		I was given extra homework to do			
Truancy	During the last 3 months of school have you skipped / bunked off school?		No<br>Yes	Ripple measure of truancy	Binary y/n
E-cigarette use	Which of the following best describes you?		I currently smoke e cigarettes<br>I have tried e cigarettes in the past 12 months but do not currently smoke them	New	Binary – ever/ never

For peer review only

		I have tried e cigarettes longer than 12 months ago but do not currently smoke them<br>I have never tried e-cigarettes		
Perceived lack of school safety	Do you feel safe at this school?	All of the time<br>Most of the time Some of the time<br>Never	HSE measure of school safety	Binary – some of the time/never versus other options

For peer review only

For peer review only - http://bmjopen.bmj.com/site/about/guidelines.xhtml

As with our analysis of primary and secondary outcomes, our analyses of outcomes in this paper were intention-to-treat, including all schools and participants at each wave. Each outcome measure was analysed using a separate mixed-model with the measure from each time point treated as a repeated measures. Fixed effects of time (baseline, 24 months and 36 months) and the interaction between arm and time were specified, and estimated baseline measures were constrained to be identical in the two arms of the trial. This is equivalent to adjusting for baseline but enables data from all participants to contribute to the analysis, even where there are missing data at follow up. We specified random effects for school and participants, to allow for correlations within schools and repeated measures within participants. We undertook analyses adjusted for baseline measures of outcomes, sex, ethnicity, socioeconomic status (Family Affluence Scale, FAS) as well as for the school stratifying factors.

We used appropriate multilevel models to examine the effects of the intervention. For quantitative measures, we used mixed linear-regression models with random effects at the level of participants and schools to estimate adjusted mean differences (MD) between arms. For binary outcomes, we used mixed effects logistic regression models, with random effects for schools and indiviuals, reporting unadjusted and adjusted odds ratios (OR). Evidence for any moderation of intervention effects on our outcomes by student sex and socioeconomic status (Family Affluence Scale) was assessed by Wald tests for the treatment by subgroup interaction terms. We also calculated Cronbach's alphas to assess the inter-item reliability of our multi-question measures of student perpetration of aggressive behaviours in or outside school and student participation in school disciplinary procedures.

The trial was prospectively registered as ISRCTN10751359 with the ISRCTN Registry on 30/01/2014, and approved by the UCL Ethics Committee (ref 5248/001). Informed consent was sought from head-teachers for randomisation and intervention, and from students, deemed competent by schools to do so, for participation in surveys. Parents were informed about the study and could withdraw their children from research activities.

For peer review only - http://bmjopen.bmj.com/site/about/guidelines.xhtml

RESULTS

In total, 6,667 students in the 40 schools provided data at baseline, representing a participation rate of 93.6% of registered students (92.9% in intervention arm; 94.3% control arm). Student characteristics and baseline reports of the outcomes examined in this paper are reported in table 3, with good balance between arms.

TABLE 3: CHARACTERISTICS OF SCHOOLS AND STUDENTS AT BASELINE BY TRIAL ARM

**School characteristics**	**Control 20 schools**	**Intervention 20 schools**	**Overall 40 schools**
School sex mix, n (%)			
Mixed	15 (75·0)	15 (75·0)	30 (75·0)
Girls	3 (15·0)	4 (20·0)	7 (17·5)
Boys	2 (10·0)	1 (5·0)	3 (7·5)
Ofsted rating*, n (%)			
Excellent	5 (25·0)	6 (30·0)	11 (27·5)
Good	13 (65·0)	12 (60·0)	25 (62·5)
Requires improvement	2 (10·0)	2 (10·0)	4 (10·0)
Value added score, mean (SD)	1,003 (24·8)	1,004 (20·4)	1,003 (22·4)
Proportion of students on free school means, mean (SD)	36 (18·0)	35 (22·0)	36 (20·0)
Income Deprivation Affecting Children Index (IDACI), mean (SD)	0·26 (0·2)	0·24 (0·2)	0·25 (0·2)
**Student socio-demographic characteristics**	**3,347 students†**	**3,320 students†**	**6,667 students†**
Age, mean (SD)	12 (0·4)	12 (0·4)	12 (0·4)
Sex, n (%)			
Male	1,639 (49·9)	1,464 (44·9)	3,103 (47·3)
Female	1,649 (50·2)	1,804 (55·2)	3,453 (52·7)
Ethnicity, n (%)			
White British	1,391 (41·5)	1,221 (37·3)	2,612 (39·7)
White other	291 (8·8)	273 (8·3)	564 (8·6)
Asian/Asian British	859 (25·9)	786 (24·0)	1,645 (25·0)
Black/Black British	384 (11·6)	535 (16·4)	919 (14·0)
Chinese/Chinese British	11 (0·3)	35 (1·1)	46 (0·7)
Mixed ethnicity	238 (7·2)	224 (6·9)	462 (7·0)
Other	140 (4·2)	198 (6·1)	338 (5·1)
Family affluence scale, mean (SD)	6 (1·8)	6 (1·8)	6 (1·8)
**Student baseline rates of outcomes**			
Cyber-bullying perpetration, n (%)	290 (8·9)	279 (8·6)	569 (8·7)
Cyber-bullying victimisation, n (%)	522 (16·0)	467 (14·5)	989 (15·3)
Truancy, n (%)	189 (5·9)	182 (5·8)	371 (5·8)
E-cigarette use, n (%)	187 (5·8)	131 (4·2)	318 (5·0)
Perceived lack of school safety, n (%)	493 (15·6)	440 (14·5)	933 (15·1)
Student-reported observations of other students perpetrating aggressive behaviours at school, mean (SD)	2·30 (1·61)	2·04 (1·65)	2·17 (1·64)

For peer review only - http://bmjopen.bmj.com/site/about/guidelines.xhtml

For peer review only

School characteristics	Control	Intervention	Overall
Perpetration of aggressive behaviour in/outside school, mean (SD)	0·06 (0·31)	0·06 (0·33)	0·06 (0·32)
Participation in school disciplinary procedures, mean (SD)	2·47 (2·96)	2·39 (3·00)	2·43 (2·98)

* One control school did not have an Ofsted rating
† The number of students who responded at this survey; actual number of responses to each question varies, but item non-response is similar across arms.

All schools participated in the surveys at 24 and 36 months, with student participation rates being similar by arms (figure 1).

Cronbach's alphas for our ESYTC measures of student perpetration of aggressive behaviours in or outside school was 0.55 and for our ESYTC measure of student participation in school disciplinary procedures was 0.79.

Our broader student outcomes at 24 and 36 months are reported in table 4. At 24 months comparing intervention with control schools, we found lower rates of: cyber-bullying victimisation (OR=0.77; 95% confidence interval (CI) 0.61, 0.98; P=0.035) but not perpetration; and e-cigarette use (OR=0.60 95% CI 0.43, 0.83; P=0.002). Students in intervention schools were more likely to report lack of perceived school safety at 24 months than controls (OR=1.38, 95% CI 1.10, 1.75; P=0.006). There was no evidence of difference between arms in: student-reported observations of other students perpetrating aggressive behaviours at school; perpetration of aggressive behaviours in or outside school; or truancy. There was weak to moderate evidence of lower reported participation in school disciplinary procedures in intervention compared with control schools at 24 months (MD=-0.16, 95% CI -0.32, 0.00; P=0.043).

For peer review only - http://bmjopen.bmj.com/site/about/guidelines.xhtml

TABLE 4: OUTCOMES AT 24- AND 36-MONTH FOLLOW-UP

**Outcomes**	**24 months**				**36 months**			
	**Control n/N (%) or mean (SD), N**	**Intervention n/N (%) or mean (SD), N (%)**	**Unadjusted OR or MD (95% confidence interval), P-value**	**Adjusted OR or MD (95% confidence interval), P-value**	**Control n/N (%) or mean (SD)**	**Intervention n/N (%) or mean (SD), N (%)**	**Unadjusted OR or MD (95% confidence interval), P-value**	**Adjusted OR or MD (95% confidence interval), P-value**
Cyber-bullying victimisation	443/3116 (14.2%)	340/2993 (11.4%)	OR=0.79 (0.63 to 0.99), 0.041	OR= 0.77 (0.61 to 0.98), 0.035	347/2987 (11.6%)	266/2754 (9.7%)	OR=0.83 (0.65 to 1.05), 0.121	OR=0.80 (0.62 to 1.05), 0.110
Cyber-bullying perpetration	257/3112 (8.3%)	229/2984 (7.7%)	OR=0.93 (0.71 to 1.21), 0.572	OR=0.90 (0.67 to 1.19), 0.450	287/3008 (9.5%)	193/2766 (7.0%)	OR=0.66 (0.51 to 0.87), 0.003	OR=0.65 (0.48 to 0.88), 0.005
Perceived lack of school safety	543/3106 (17.5%)	568/2944 (7.7%)	OR=1.39 (1.11 to 1.74), 0.004	OR=1.39 (1.10 to 1.75), 0.006	601/2919 (20.6%)	532/2682 (19.8%)	OR=1.08 (0.86 to 1.36), 0.495	OR=1.05 (0.83 to 1.34), 0.440
Student-reported observations of other students perpetrating aggressive behaviours at school	2.43 (1.65), 3179	2.12 (1.71), 3063	MD=-0.05 (-0.14 to 0.05), 0.344	MD=-0.08 (-0.18 to 0.01), 0.096	2.23 (1.77), 3069	2.07 (1.76), 2834	MD=0.11 (0.01 to 0.20), 0.030	MD=0.10 (0.00 to 0.20), 0.049
Perpetration of aggressive behaviours in/ outside school	0.10 (0.44), 3149	0.08 (0.39), 3017	MD=-0.014 (-0.037 to 0.009), 0.229	MD=-0.009 (-0.034 to 0.015), 0.456	0.12 (0.50), 3023	0.09 (0.43), 2778	MD=-0.030 (-0.054 to -0.007), 0.012	MD=-0.031 (-0.056 to -0.006), 0.016
Participation in school disciplinary procedures	2.74 (3.28), 3128	2.36 (3.12), 3009	MD=-0.222 (-0.375 to -0.069), 0.004	MD=-0.160 (-0.320 to 0), 0.043	2.55 (3.21), 3017	2.15 (2.94), 2757	MD=-0.326 (-0.484 to -0.169), <0.001	MD=-0.320 (-0.480 to -0.150), <0.001
Truancy	340/3088 (11.0%)	299/2961 (10.1%)	OR=0.93 (0.72 to 1.20), 0.582	OR=0.92 (0.70 to 1.21), 0.551	426/2981 (14.3%)	294/2750 (10.7%)	OR=0.66 (0.51 to 0.85), 0.001	OR=0.64 (0.49 to 0.84), 0.001

For peer review only - http://bmjopen.bmj.com/site/about/guidelines.xhtml

E-cigarette use	558/3061 (18.2%)	352/2913 (12.1%)	OR=0.62 (0.45 to 0.86), 0.004	OR=0.60 (0.43 to 0.83), 0.002	630/2946 (21.4%)	394/2692 (14.6%)	OR=0.60 (0.44 to 0.83), 0.002	OR=0.59 (0.42 to 0.82), 0.002

For peer review only

At 36 months comparing intervention and control schools, we found reduced rates of: cyber-bullying perpetration (OR=0.65, 95% CI 0.48, 0.88; P=0.005) but not victimisation; perpetration of aggression in or outside school (MD=-0.031 95% CI -0.056, -0.006; P=0.016); participation in school disciplinary procedures (MD=-0.320, 95% CI -0.480, -0.150; P<0.001); truancy (OR=0.64, 95% CI 0.49, 0.84; P=0.001); and e-cigarette use (OR=0.59, 95% CI 0.42, 0.82; P=0.002). There was weak to moderate evidence of lower student-reported observations of other students perpetrating aggressive behaviours at school (MD=0.10, 95% CI 0.00, 0.20; P=0.049). There were no evidence of difference in perceived school safety.

Table 5 presents outcomes and follow-up points for which there was evidence of moderation. We found evidence that intervention effects on cyber-bullying perpetration at 24 months were moderated by student sex, such that effects were larger for boys (P=0.002). Intervention effects on observed aggression by other students at 24 months were moderated by student sex and socioeconomic status, with effects larger for girls (P=0.02) and affluent students (P=0.03). Effects on perceived lack of school safety at 24 months were larger for girls (P=0.001), and at 36 months were larger for students from poorer families (P=0.002). Effects on participations in school disciplinary procedures at 24 and 36 months were larger for boys (P<0.001; P=0.001 respectively). Effects on truancy at 24 months were larger for boys (P=0.015). Effects on e-cigarette use at 24 and 36 months were larger for boys (P=0.014; P<0.001 respectively).

TABLE 5: MODERATION

**Outcome**	**Follow-up (months) where evidence of moderation**	**Moderator**		**Association (95% CI)**	**Interaction P value**
		Variable	***Categories***		
Perpetration cyber-bullying	24	Sex	Boys	OR=0.61 (0.41, 0.89)	0.002
			Girls	1.19 (0.85, 1.67)	
Observed aggression	24	Sex	Boys	MD=0.01 (-0.11, 0.13)	0.02
			Girls	MD=-0.15 (-0.26, -0.04)	

For peer review only - http://bmjopen.bmj.com/site/about/guidelines.xhtml

		Family affluence	Low	MD=-0.18 (-0.62, 0.25)	0.03
			Middle	MD=0.04 (-0.09, 0.18)	
			High	MD=-0.14 (-0.25, -0.03)	
Lack of safety	24	Sex	Boys	OR=0.99 (0.73, 1.35)	0.001
			Girls	OR=1.74 (1.33, 2.27)	
	36	Family affluence	Low	OR=3.07 (0.99-9.54)	0.002
			Middle	OR=0.72 (0.51, 1.01)	
			High	OR=1.23 (0.94, 1.60)	
Participation in school disciplinary procedures	24	Sex	Boys	MD=-0.39 (-0.60, -0.19)	<0.001
			Girls	MD=0.02 (-0.17, 0.20)	
	36	Sex	Boys	MD=-0.55 (-0.76, -0.34)	0.001
			Girls	MD=-0.14 (-0.33, 0.05)	
Truancy	24	Sex	Boys	OR=0.69 (0.48, 0.99)	0.015
			Girls	OR=1.13 (0.83-1.55)	
E-cigarette use	24	Sex	Boys	OR=0.45 (0.30, 0.67)	0.014
			Girls	OR=0.80 (0.54, 1.20	
	36		Boys	OR=0.35 (0.23, 0.53	<0.001
			Girls	OR=0.94 (0.63, 1.40).	

For peer review only

DISCUSSION

Summary of key findings

We report an analysis of broader system impacts on student health and school functioning outcomes of this settings-based intervention aim to render schools more health-promoting environments, which

was previously reported to be effective in reducing bullying victimisation and use of alcohol, tobacco and drugs, as well as promoting mental and physical health.(1) We found evidence at 36 but not 24 months of intervention effects on: cyber-bullying perpetration; student observations of aggression by other students; students' own perpetration of aggressive behaviours in or outside school; truancy; and participation in school disciplinary procedures. There was evidence of an effect on increased student perceptions of lack of school safety at 24 but not 36 months. There was also evidence of an effect at 24 but not 36 months on cyber-bullying victimisation. We found evidence of an effect on e-cigarette use at both time-points.

These findings suggest that our intervention, which aimed to reduce bullying via making schools more engaging environments, and which did not explicitly focus on cyber-bullying, might nonetheless have been effective in reducing this. Our findings also suggest that the intervention might, contrary to the main analyses, have reduced rates of aggression including aggression beyond the school environment. However, results across time-points and measures are somewhat inconsistent, probably as a result of chance. Our intervention also appears to have reduced student use of e-cigarettes, which is an important finding given increasing concerns about this as a gateway to tobacco use.(23) This evidence of additional health impacts provides further evidence in support of the intervention theory of change, that it is possible to improve young people's health across a range of areas by addressing the school as a potentially salutogenic environment rather than merely as a setting for individual-focused health education in classrooms. Furthermore, our finding of broader impacts on school functioning in terms of reduced truancy and student involvement in discipline systems, provides evidence of the knock-on consequences of a health intervention disrupting the school system to achieve impacts in the domain of education.

Intervention effects were moderated in some cases by student sex and family affluence. Effects were larger for boys regarding reduced cyber-bullying perpetration and truancy at 24 months, and reduced participations in school disciplinary procedures and e-cigarette use at 24 and 36. For girls,

For peer review only - http://bmjopen.bmj.com/site/about/guidelines.xhtml

intervention effects were greater regarding reduced observed aggression by other students and decreased perceived school safety at 24 months. Effects for decreased perceived school safety at 36 months were also larger for students from poorer families. Effects on reduced observed aggression at 24 months were larger for affluent students. These findings contrast with moderator analyses for our primary and secondary outcomes, where benefits were generally larger for boys and no different for those from poorer families.(1)

Our finding of increased student perceptions of lack of safety in intervention versus control schools at 24 months, and among poorer students at 36 months, is of concern. We note that this association runs counter to our previous findings for actual rates of bullying victimisation(1) and to the findings in this paper on aggression. This finding may be due to chance. However, it may might be explained by the intervention's focus on bullying and aggression sensitizing students to issues of safety, leading them to feel unsafe at the initial (24 month) follow-up but with this dissipating as the intervention became normalised and exerted positive effects on bullying and aggression.

These moderator analyses add to the evidence from our main analyses that the intervention might generally have been more effective for boys than girls. As with our main trial analyses, there was less evidence for moderation by socioeconomic status. These findings of gender inequity of effects is in line with some previous research suggesting that whole-school interventions, including those to reduce violence, can sometimes be more effective for boys than girls.(1, 4) This might be because, in such interventions, violence and other problem behaviours among boys receive more attention than those experienced by girls.

Strengths and limitations

The study was a rigorously conducted experimental evaluation. These are additional analyses not included in our original protocol. Therefore, caution is required in the interpretation of significant

For peer review only - http://bmjopen.bmj.com/site/about/guidelines.xhtml

findings. However, the analyses are guided by explicit, theory-driven hypotheses, as set out in our introduction, rather than being the product of subjecting all measures to analysis and merely reporting significant findings. We relied on student self-reports as these were less likely than school routine data to be biased by delivery of the intervention. Where possible, we used reliable existing measures. While our measure of student participation in disciplinary procedures had high inter-item reliability, this was lower for our measure of student perpetration of aggressive behaviours in or outside school so our conclusions regarding effects on this outcome should be cautious.

Implications for policy and research

Our findings suggest that the intervention disrupted school systems to achieve a range of unintended but beneficial impacts on student health and school functioning not captured in the main trial analyses.(2, 3) These results suggest that it is possible to achieve public health improvements across a range of outcomes using a single coordinated intervention which focuses on environmental transformation rather than individual behaviour-change. This is important given the impracticality of implementing different interventions for multiple outcomes in schools.(21) Our findings also suggest important benefits for education and school functioning. It appears that the intervention's previously reported effects on reducing bullying victimisation and improving student commitment to school(1, 22) translated into reduced student truancy and participation in school disciplinary procedures. This is especially important evidence for school leaders suggesting the potential educational benefits of whole-school health interventions. Our analyses suggest this intervention worked more effectively for boys than girls. Further research is needed into how to ensure school-based interventions are more equitable, perhaps by ensuring these address less overt forms of student disengagement and conflict.

Figure 1: Trial participants

ACKNOWLEDGEMENTS

For peer review only

For peer review only - http://bmjopen.bmj.com/site/about/guidelines.xhtml

We are grateful to the staff and students of participating schools for their dedication to the intervention and completion of the outcome surveys and process evaluation surveys and interviews. We are very grateful for the advice and support of our Trial Steering Committee and Data Monitoring Committee. All those who contributed significantly to this work are listed in these acknowledgements.

COMPETING INTERESTS

None to declare.

This article has not been submitted and will not be submitted for publication elsewhere while under consideration by BMJ Open. The paper includes data (on response rates and baseline comparisons between intervention and control groups) that was included in the paper cited below. Other analyses are new to this paper.

Bonell C, Allen E, Warren E, McGowan J, Bevilacqua L, Jamal F, et al. Initiating change in the school environment to reduce bullying and aggression: a cluster randomised controlled trial of the Learning Together (LT) intervention in English secondary schools. The Lancet. 2018;392(10163):2452-64.

FUNDING

This work was supported by the National Institute for Health Research (NIHR) in England under its Public Health Research Board (12/153/60) and the Education Endowment Foundation (no grant number). The views expressed in this publication are those of the authors and do not necessarily reflect those of the National Health Service (NHS), the NIHR or the Department of Health for England. The study funders (National Institute for Health Research and Education Endowment

For peer review only - http://bmjopen.bmj.com/site/about/guidelines.xhtml

Foundation played no role in the: study; collection, analysis and interpretation of data; the writing of the report; or the decision to submit the manuscript for publication.

DATA STATEMENT

Anonymised data from the study are available on request.

For peer review only

For peer review only - http://bmjopen.bmj.com/site/about/guidelines.xhtml

REFERENCES

1. Bonell C, Allen E, Warren E, McGowan J, Bevilacqua L, Jamal F, et al. Initiating change in the school environment to reduce bullying and aggression: a cluster randomised controlled trial of the Learning Together (LT) intervention in English secondary schools. The Lancet. 2018;392(10163):2452-64.
2. Moore GF, Evans RE, Hawkins J, Littlecott H, Melendez-Torres GJ, Bonell GJ, et al. From complex social interventions to interventions in complex social systems: future directions and unresolved questions for intervention development and evaluation. Evaluation and Program Planning. 2019;;25(1):23-45.
3. Rosas SR. Systems thinking and complexity: considerations for health promoting schools Health Promotion International. 2017;32(2):301-11.
4. Flay BR, Graumlich S, Segawa E, Burns JL, Holliday MY. Effects of 2 prevention programs on high-risk behaviors among African American youth: a randomized trial. Archives of Pediatrics & Adolescent Medicine. 2004;158(4):377-84.
5. Langford R, Bonell CP, Jones HE, Pouliou T, Murphy SM, Waters E, et al. The WHO Health Promoting School framework for improving the health and well-being of students and staff. Cochrane Database of Systematic Reviews 2011, Issue 1 Art No: CD008958. 2014.
6. Patton G, Bond L, Carlin JB, Thomas L, Butler H, Glover S, et al. Promoting social inclusion in schools: group-randomized trial of effects on student health risk behaviour and well-being. American Journal of Public Health. 2006;96(9):1582-7.
7. Bonell C, Allen E, Christie D, Elbourne D, Fletcher A, Grieve R, et al. Initiating change locally in bullying and aggression through the school environment (INCLUSIVE): study protocol for a cluster randomised controlled trial. Trials. 2014;15:381.
8. Morrison B. Restorative justice in schools. In: Elliot E, Gordon RM, editors. New directions in restorative justice: Issues, practices, evaluation. Cullompton, UK: Willan Publishing; 2005. p. 26-52.
9. Markham WA, Aveyard P. A new theory of health promoting schools based on human functioning, school organisation and pedagogic practice. Social Science & Medicine. 2003;56(6):1209-20.
10. Bond L, Wolfe S, Tollit M, Butler H, Patton G. A comparison of the Gatehouse Bullying Scale and the peer relations questionnaire for students in secondary school. Journal of School Health. 2007;77(2):75-9.
11. Inchley J, Currie D, Young T, Samdal O, Torsheim T, Augustson L, et al. Growing up unequal: gender and socioeconomic differences in young people's health and well-being. Copenhagen: WHO Europe, 2016 Contract No.: ISBN 978 92 890 5136 1.
12. Arseneault L. Annual Research Review: The persistent and pervasive impact of being bullied in childhood and adolescence: implications for policy and practice. J Child Psychol Psychiatry. 2017.
13. Hair EC, Park MJ, Ling TJ, Moore KA. Risky behaviors in late adolescence: co-occurrence, predictors, and consequences. J Adolesc Health. 2009;45(3):253-61.
14. Copeland WE, Wolke D, Angold A, Costello EJ. Adult psychiatric outcomes of bullying and being bullied by peers in childhood and adolescence. JAMA Psychiatry. 2013;70(4):419-26.
15. Lereya ST, Copeland WE, Costello EJ, Wolke D. Adult mental health consequences of peer bullying and maltreatment in childhood: two cohorts in two countries. Lancet Psychiatry. 2015;2(6):524-31.
16. Fisher HL, Moffitt TE, Houts RM, Belsky DW, Arseneault L, Caspi A. Bullying victimisation and risk of self harm in early adolescence: longitudinal cohort study. BMJ. 2012;344:e2683.
17. Winsper C, Lereya T, Zanarini M, Wolke D. Involvement in bullying and suicide-related behavior at 11 years: a prospective birth cohort study. J Am Acad Child Adolesc Psychiatry. 2012;51(3):271-82 e3.
18. Fantaguzzi C, Allen E, Miners A, Christie D, Opondo C, Sadique Z, et al. Health-related quality of life associated with bullying and aggression: a cross-sectional study in English secondary schools. Eur J Health Econ. 2017.
19. Bevilacqua L, Shackleton N, Hale D, Allen E, Bond L, Christie D, et al. The role of family and school-level factors in bullying and cyberbullying: a cross-sectional study. BMC Pediatr. 2017;17(1):160.
20. Smith DJ. School experience and delinquency at ages 13 to 16. Edinburgh: Centre for Law and Society, University of Edinburgh; 2006.
21. Bonell C, Humphrey N, Fletcher A, Moore L, Anderson R, Campbell R. Why schools should promote students' health and wellbeing Education policy shouldn't focus solely on academic attainment. British Medical Journal. 2014;348:g3078.
22. Bonell C, Allen E, Opondo C, Warren E, Elbourne D, Sturgess J, et al. Examining intervention mechanisms of action using mediation analysis within a randomised trial of a whole-school health intervention. Journal of Epidemiology and Community Health http://dxdoiorg/101136/jech-2018-211443. 2019.

For peer review only - http://bmjopen.bmj.com/site/about/guidelines.xhtml

23. Barrington-Trimis JL, Kong G, Leventhal AM, Liu F, Mayer M, Boley Cruz T, et al. E-cigarette use and subsequent smoking frequency among adolescents. Pediatrics. 2018;142(6):e20180486.
24. Smith PK, Mahdavi J, Carvalho M, Fisher S, Russell S, Tippett N. Cyberbullying: Its nature and impact in secondary school pupils. Journal of Child Psychology and Psychiatry. 2008;49(376):e385.
25. Bonell C, Sorhaindo A, Allen E, Strange V, Wiggins M, Fletcher A, et al. Pilot multi-method trial of a school-ethos intervention to reduce substance use: building hypotheses about upstream pathways to prevention. Journal of Adolescent Health. 2010;47(6):555-63.
26. Stephenson JM, Strange V, Forrest S, Oakley A, Copas A, Allen E, et al. Pupil-led sex education in England (RIPPLE study): cluster-randomised intervention trial. Lancet. 2004;364(9431):338-46.

For peer review only

For peer review only - http://bmjopen.bmj.com/site/about/guidelines.xhtml

FIGURE 1: TRIAL PARTICIPANTS

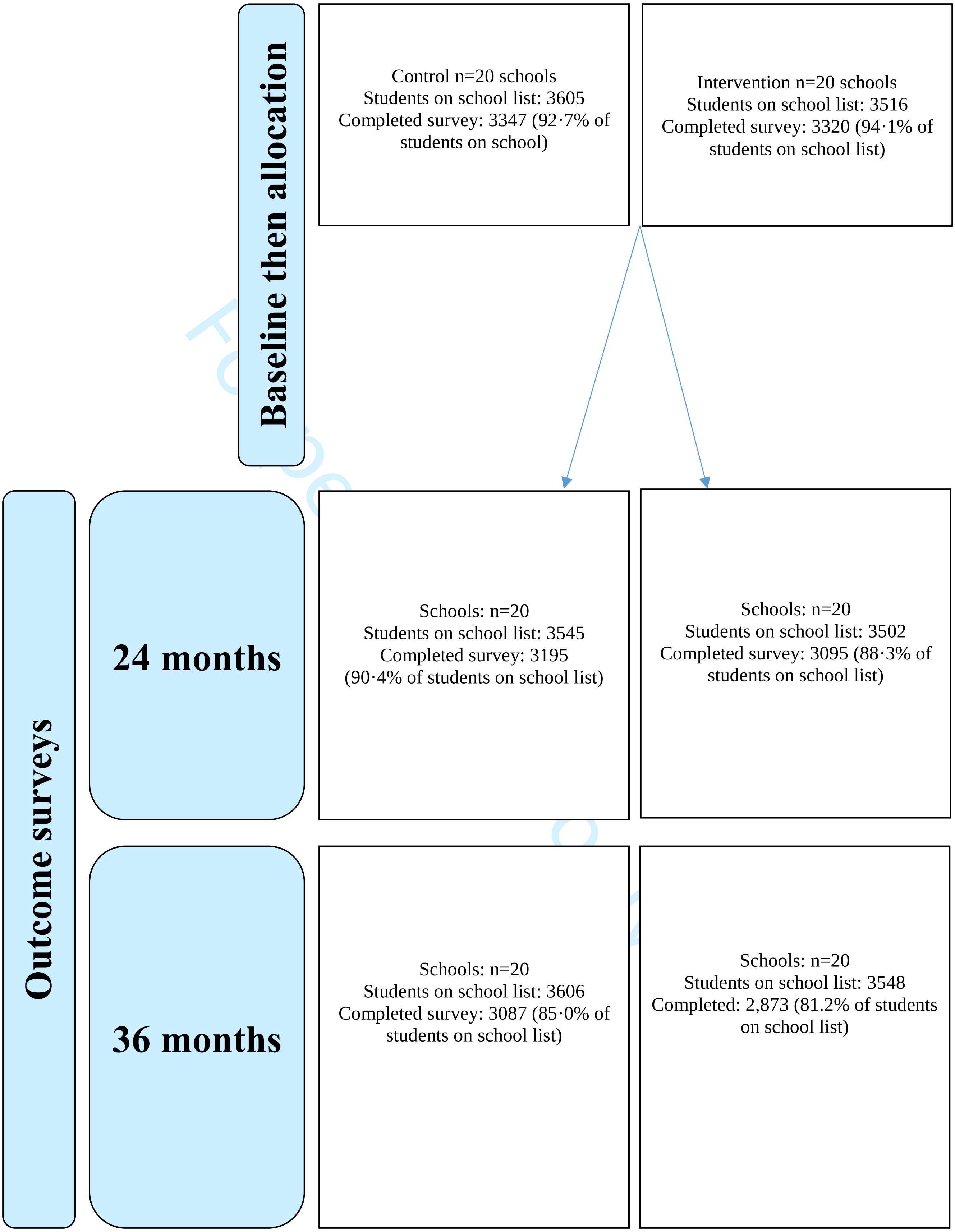

CONSORT 2010 checklist of information to include when reporting a randomised trial*

Section/Topic	Item No	Checklist item	Reported on page No
**Title and abstract**			
	1a	Identification as a randomised trial in the title	1,3,5
	1b	Structured summary of trial design, methods, results, and conclusions (for specific guidance see CONSORT for abstracts)	3,4
**Introduction**			
Background and objectives	2a	Scientific background and explanation of rationale	5-8
	2b	Specific objectives or hypotheses	8
**Methods**			
Trial design	3a	Description of trial design (such as parallel, factorial) including allocation ratio	8
	3b	Important changes to methods after trial commencement (such as eligibility criteria), with reasons	NA
Participants	4a	Eligibility criteria for participants	8,9
	4b	Settings and locations where the data were collected	9
Interventions	5	The interventions for each group with sufficient details to allow replication, including how and when they were actually administered	9
Outcomes	6a	Completely defined pre-specified primary and secondary outcome measures, including how and when they were assessed	Additional analysis. 9-13
	6b	Any changes to trial outcomes after the trial commenced, with reasons	NA
Sample size	7a	How sample size was determined	reference to main trial report
	7b	When applicable, explanation of any interim analyses and stopping guidelines	NA
Randomisation:			9 + reference to main trial report
Sequence generation	8a	Method used to generate the random allocation sequence	
	8b	Type of randomisation; details of any restriction (such as blocking and block size)	9
Allocation concealment	9	Mechanism used to implement the random allocation sequence (such as sequentially numbered containers), describing any steps taken to conceal the sequence until interventions were assigned	9 + reference to main trial

For peer review only

mechanism			report
Implementation	10	Who generated the random allocation sequence, who enrolled participants, and who assigned participants to interventions	9 + reference to main trial report
Blinding	11a	If done, who was blinded after assignment to interventions (for example, participants, care providers, those assessing outcomes) and how	9
	11b	If relevant, description of the similarity of interventions	NA
Statistical methods	12a	Statistical methods used to compare groups for primary and secondary outcomes	14 + reference to main trial report
	12b	Methods for additional analyses, such as subgroup analyses and adjusted analyses	14
**Results**			
Participant flow (a diagram is strongly recommended)	13a	For each group, the numbers of participants who were randomly assigned, received intended treatment, and were analysed for the primary outcome	15, figure 1 + reference to main trial report
	13b	For each group, losses and exclusions after randomisation, together with reasons	15, 16 + reference to main trial report
Recruitment	14a	Dates defining the periods of recruitment and follow-up	9
	14b	Why the trial ended or was stopped	NA
Baseline data	15	A table showing baseline demographic and clinical characteristics for each group	Table 2
Numbers analysed	16	For each group, number of participants (denominator) included in each analysis and whether the analysis was by original assigned groups	Tables 3-4
Outcomes and estimation	17a	For each primary and secondary outcome, results for each group, and the estimated effect size and its precision (such as 95% confidence interval)	Table 3
	17b	For binary outcomes, presentation of both absolute and relative effect sizes is recommended	reference to main trial report
Ancillary analyses	18	Results of any other analyses performed, including subgroup analyses and adjusted analyses, distinguishing pre-specified from exploratory	Table 3

For peer review only - http://bmjopen.bmj.com/site/about/guidelines.xhtml
For peer review only

Harms	19	All important harms or unintended effects in each group (for specific guidance see CONSORT for harms)	reference to main trial report
**Discussion**			
Limitations	20	Trial limitations, addressing sources of potential bias, imprecision, and, if relevant, multiplicity of analyses	23
Generalisability	21	Generalisability (external validity, applicability) of the trial findings	23
Interpretation	22	Interpretation consistent with results, balancing benefits and harms, and considering other relevant evidence	23
**Other information**			
Registration	23	Registration number and name of trial registry	14
Protocol	24	Where the full trial protocol can be accessed, if available	14
Funding	25	Sources of funding and other support (such as supply of drugs), role of funders	24-25

*We strongly recommend reading this statement in conjunction with the CONSORT 2010 Explanation and Elaboration for important clarifications on all the items. If relevant, we also recommend reading CONSORT extensions for cluster randomised trials, non-inferiority and equivalence trials, non-pharmacological treatments, herbal interventions, and pragmatic trials. Additional extensions are forthcoming: for those and for up to date references relevant to this checklist, see www.consort-statement.org.

For peer review only

For peer review only - http://bmjopen.bmj.com/site/about/guidelines.xhtml
